# A gain-of-function single nucleotide variant creates a new promoter which acts as an orientation-dependent enhancer-blocker

**DOI:** 10.1038/s41467-021-23980-6

**Published:** 2021-06-21

**Authors:** Yavor K. Bozhilov, Damien J. Downes, Jelena Telenius, A. Marieke Oudelaar, Emmanuel N. Olivier, Joanne C. Mountford, Jim R. Hughes, Richard J. Gibbons, Douglas R. Higgs

**Affiliations:** 1 MRC Weatherall Institute of Molecular Medicine, University of Oxford, Oxford, UK; 2 MRC Molecular Haematology Unit, MRC Weatherall Institute of Molecular Medicine, University of Oxford, Oxford, UK; 3 MRC WIMM Centre for Computational Biology, MRC Weatherall Institute of Molecular Medicine, Radcliffe Department of Medicine, University of Oxford, Oxford, UK; 4 The Max Planck Institute for Biophysical Chemistry, Göttingen, Germany; 5 Albert Einstein College of Medicine, Department of Cell Biology, New York, NY USA; 6 Institute of Cardiovascular and Medical Sciences, University of Glasgow, Glasgow, UK; 7 Scottish National Blood Transfusion Service, University of Glasgow, Glasgow, UK

**Keywords:** Gene expression, Gene regulation, Epigenetics

## Abstract

Many single nucleotide variants (SNVs) associated with human traits and genetic diseases are thought to alter the activity of existing regulatory elements. Some SNVs may also create entirely new regulatory elements which change gene expression, but the mechanism by which they do so is largely unknown. Here we show that a single base change in an otherwise unremarkable region of the human α-globin cluster creates an entirely new promoter and an associated unidirectional transcript. This SNV downregulates α-globin expression causing α-thalassaemia. Of note, the new promoter lying between the α-globin genes and their associated super-enhancer disrupts their interaction in an orientation-dependent manner. Together these observations show how both the order and orientation of the fundamental elements of the genome determine patterns of gene expression and support the concept that active genes may act to disrupt enhancer-promoter interactions in mammals as in Drosophila. Finally, these findings should prompt others to fully evaluate SNVs lying outside of known regulatory elements as causing changes in gene expression by creating new regulatory elements.

## Abstract (web summary)

The role of promoters as potential insulator elements has been largely unexplored in mammals. Here the authors show that a single nucleotide variant in the α-globin locus forms a new promoter and acts as an orientation-dependent enhancer-blocking insulator element.

## Introduction

A large proportion of the single nucleotide variants (SNVs) associated with human traits and predisposition to human disease lie within non-coding regions of the genome^1^. Therefore, the causative variants are presumed to affect the fundamental regulatory elements of the genome including enhancers, promoters and boundary elements^2,3^. We previously described the emergence of a new transcriptional unit in a non-regulatory region of the α-globin locus which causes downregulation of α-globin expression (α-thalassaemia) in individuals from Melanesia. In the same study we identified a candidate for the causal mutation: a single nucleotide (T to C) change which resulted in a new binding site for the erythroid master regulator GATA-1 and the production of a new RNA transcript^4^. This provides an example of a trait associated SNV that appears to create a new regulatory element rather than disrupting an existing element. Few “gain-of-function” SNVs have been identified^4–7^ and as yet the prevalence and full repertoire of mechanisms by which such SNVs might alter gene expression have not yet been determined.

The candidate SNV (a T to C transition at coordinate hg19 chr16:209,709) which is thought to down regulate α-globin expression is found within an unremarkable non-coding region of the α-globin locus. This multi gene cluster lies within a relatively small, well characterised topologically associated sub-domain (a sub-TAD of ~80 kb) flanked by CTCF-boundary elements within the telomeric region of chromosome 16^8–11^. The sub-TAD includes embryonic (ζ - *HBZ*), fetal/adult (α–*HBA1/2*) and theta (θ–*HBQ*) globin genes which are regulated by four enhancers (R1, R2, R3 and R4) arranged in the order 5′-R1-R2-R3-R4-ζ-α2-α1-θ1-3′. The locus is only active in erythroid cells where active regulatory regions are found in open chromatin associated with acetylated lysine at position 27 of histone H3 (H3K27ac). Enhancers are marked by monomethyl lysine 4 of histone H3 (H3K4me1) and promoters by trimethyl lysine 4 of histone H3 (H3K4me3). Although we have previously shown that the emergence of a new site of transcriptional activity is associated with downregulation of α-globin expression^4^, whether the T to C change is causative and the mechanism by which this disrupts normal regulation is not yet clear.

Here we demonstrate that a point mutation in a non-regulatory region of the human α-globin locus can establish a promoter element that is capable of affecting the expression of nearby native genes. We show that that activity of the novel element cannot be easily explained by a competition model since it disrupts the native promoter-enhancer interactions in an orientation-dependent manner. Our observations underline the link between the order and orientation of the fundamental elements of the genome and highlight the complexity of gene regulation.

## Results

### Point mutation creates new promoter that downregulates native genes

**Fig. 1:**
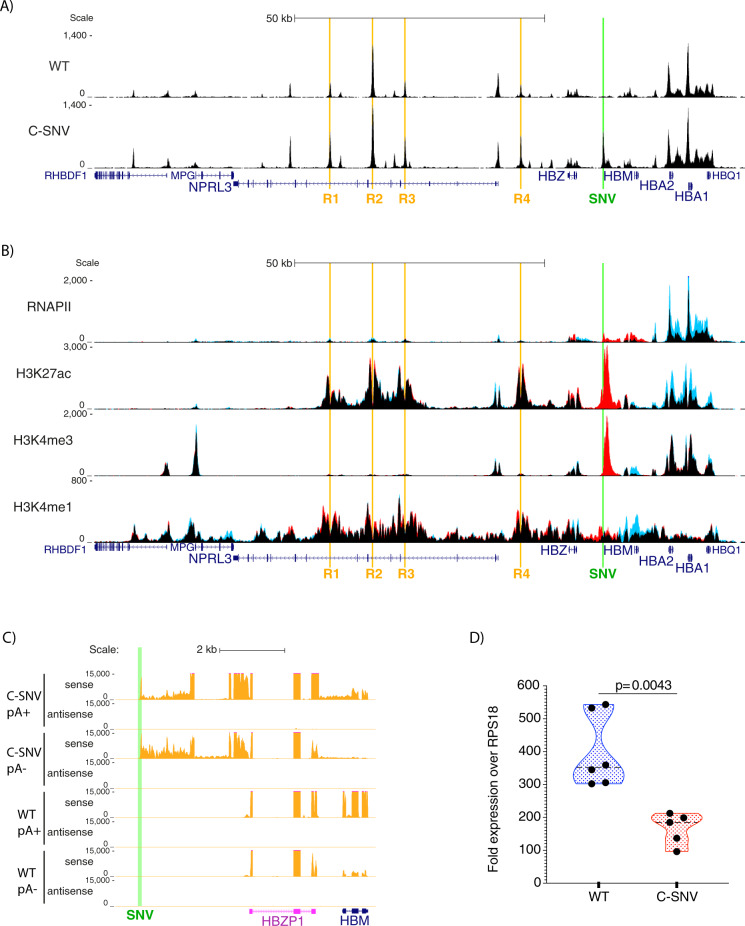
New transcriptional unit bears the marks of a unidirectional promoter and causes α-globin downregulation. **A** Chromatin accessibility in the α-globin locus as measured by ATAC-seq. The enhancer elements (R1–R4) are highlighted in orange, the site of the T to C mutation is highlighted in green (labelled SNV for single nucleotide variant), gene annotation by Refseq is in blue. Read-densities represent an average of 3 independent differentiation experiments, 3 independent wild type iPSC lines (labelled WT) or 3 iPSC clones obtained from the same patient material homozygous for the C allele of the SNV located at coordinate (hg19) chr16:209,709 (labelled C-SNV), differentiated to erythroblasts. Coordinates (hg19) chr16:108,000-238,000. **B** ChIP-seq, highlighted regions are as in **A**. Read-densities represent an average of 3 independent experiments, 3 replicates for wild type iPSC line AH017-13 (in blue) or 3 C-SNV iPSC clones obtained from the same patient material (in red) differentiated to erythroblasts. The tracks are overplayed on top of each other, black indicates shared signal while red and blue indicate signal unique for the mutant and wild type lines, respectively. Coordinates (hg19) chr16:108,000-238,000. The level of the signal in the middle of the C-SNV transcriptional unit is most likely affected by the presence of a variable number tandem repeat (inter-ζ VNTR), a 1 kb sequence in the reference genome which in reality can be much larger (over 2 kb). The artificial reduction of the reference genome means that the same signal is collapsed to a smaller length resulting in *at least* 2-fold signal increase over the middle of the region of enrichment at the C-SNV element. Since the exact sequence or size of the repeat is not known in the genomes analysed, this has not been corrected for. **C** Strand-specific RNA-seq of polyadenylated selected (pA+) and non-polyadenylated (pA−) RNA, read density (in RPKM) represents an average of 3 independent experiments, 3 replicates for wild type line AH017-13 or 3 clones of C-SNV iPSCs differentiated to erythroid cells. The region of the T to C mutation is highlighted in green (SNV), gene annotation by Refseq is in blue, pseudo genes are in pink. Coordinates (hg19) chr16:209,000–217,000. **D** qPCR quantification of *HBA1/HBA2* in reference to *RPS18* in mRNA obtained from 3 independent wild type iPSC lines (WT) or 3 iPSC clones obtained from the same patient material (C-SNV) differentiated to erythroblasts. All lines were differentiated twice (one replicate was removed as an outlier due to low levels): WT (*n* = 6) in blue, C-SNV (*n* = 5) in red. Violin plots display median (dotted black line) quartile lines (coloured dotted line) and individual data points (black dots). P-values are obtained using unpaired, two-tailed student t-test.

To investigate the SNV created by the T-C transition we generated induced pluripotent stem cell (iPSC) lines from cells that carry the C allele (C-SNV) at position 209,709 (hg19) on both copies of chromosome 16 (Supplementary Fig. 1a, b). The iPSC lines were differentiated down the erythroid pathway (Supplementary Fig. 1c)^12^. To characterise the new transcriptional unit we analysed chromatin accessibility (ATAC-seq), the epigenetic landscape (ChIP-seq) and transcription profile (RNA-seq) of the α-globin locus. In the mutant cells (C-SNV) there is a new region of open chromatin associated with the site of the candidate (T-C) mutation (Fig. 1A). This newly formed accessible site is bound by RNA polymerase II (RNAP II) and is marked by prominent peaks of H3K27ac and H3K4me3 while the difference in H3K4me1 between the wild type and the mutant C-SNV cells is far less pronounced (Fig. 1B). This chromatin signature is consistent with that observed at most active promoters^13–16^. Strand specific RNA-seq demonstrates that the C-SNV promoter produces both non-polyadenylated (pA-) and polyadenylated (pA+) transcripts, but only in the sense direction with respect to the published human genome (Fig. 1C). Thus, transcription is directed away from the enhancers, in the same direction as transcription of the α-globin genes. To determine if the mutant cells have reduced levels of α-globin mRNA, as seen in individuals carrying this mutation, we performed qPCR analysis on three mutant C-SNV iPSC clones and three independently generated normal human iPSC lines (WT) differentiated to erythroblasts. There was a roughly two-fold reduction in α-globin mRNA in the C-SNV erythroid cells compared to the wild type lines (Fig. 1D). These findings show that the iPSC system recapitulates the phenotype observed in erythroid cells from individuals carrying the C-SNV.

**Fig. 2:**
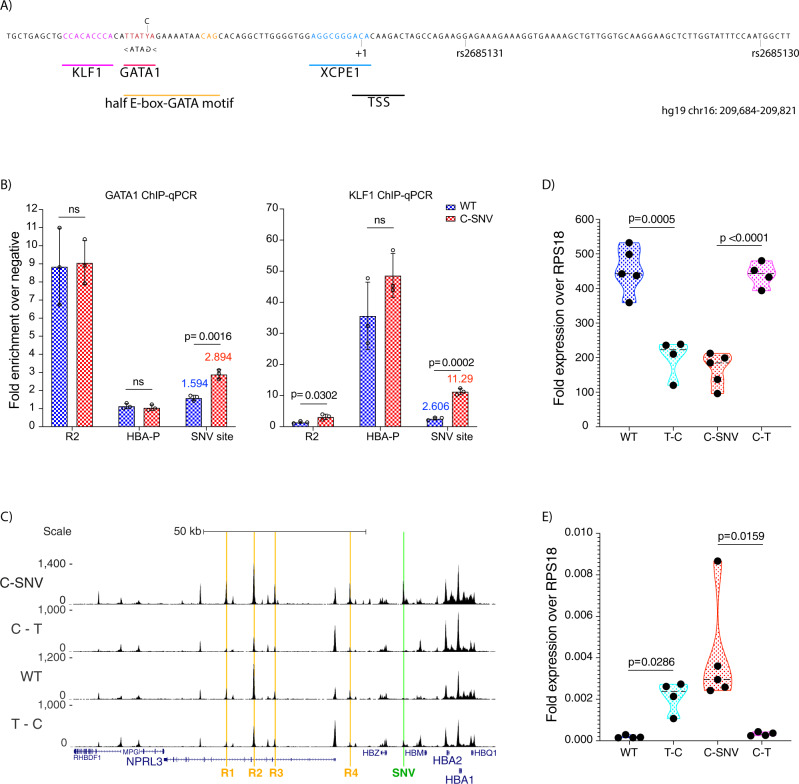
Point mutation causes the emergence of a de novo promoter. **A** 138 bp unique sequence around the causative point mutation. The SNV is labelled using the degenerate DNA symbol Y (T/C), key features are highlighted: KLF1 binding site (magenta), GATA1 (red), composite half E-box-GATA motif (orange), core promoter element XCPE1 (blue) and TSS (black). **B** ChIP-qPCR for GATA1 and KLF1, sequences for TaqMan assays can be found in Supplementary Table 1. Values represent an average of 3 independent experiments, 3 replicates for wild type iPSC line AH017-13 differentiated to erythroblasts (in blue) or 3 C-SNV iPSC clones (in red). Error bars represent one standard deviation. *P* values are obtained using unpaired, two-tailed student *t*-test. **C** Chromatin accessibility by ATAC-seq. The enhancer elements (R1–R4) are highlighted in orange, the site of the T to C mutation is highlighted in green (SNV), gene annotation by Refseq is in blue. Read-densities represent an average of 3 independent differentiation experiments: wild type iPSC line SB-AD2-01 (labelled WT), 3 C-SNV iPSC clones (labelled C-SNV), 4 clones of edited SB-AD2-01 cells where the T base at position 209,709 (hg19) of chr16 was changed to a C (labelled T–C), 4 clones of edited C-SNV iPS cells (line LA01) where the C base at position 209,709 (hg19) of chr16 was changed to a T (labelled C–T). Coordinates (hg19) chr16:108,000-238,000. **D** qPCR quantification of *HBA1/HBA2* in reference to *RPS18* in mRNA obtained from independent differentiation experiments: 5 from wild type iPSC line SB-AD2-01 (WT), 3 C-SNV iPSC clones (C-SNV) differentiated to erythroblasts twice (one replicate was removed as an outlier), 4 T–C clones (labelled T–C), 4 C–T clones (labelled C–T). WT (*n* = 5) in blue, C-SNV (*n* = 5) in red, T–C (*n* = 4) in cyan, C–T (*n* = 4) in magenta. Violin plots display median (dashed black line) quartile lines (coloured dotted line) and individual data points (black dots). *P* values are obtained using unpaired, two-tailed student *t*-test. **E** qPCR quantification of novel transcript (TaqMan assay in Supplementary Table 1) in reference to RPS18 in mRNA obtained from independent differentiation experiments as in **D**). *P* values are obtained using unpaired, two-tailed student *t*-test.

To understand how the local DNA sequence might facilitate the establishment of the novel C-SNV promoter we studied the site at the 5’ end of the transcribed region. We mapped the transcription start site (TSS) using RLM-RACE and identified the most likely transcription initiation element to be the core promoter element XCPE1^17^ (Supplementary Fig. 2). The T-C variant generates a recognition motif for the erythroid transcription factor (TF) GATA1 and this particular GATA1 site is part of a wider half E-box-GATA motif, commonly co-occupied by TAL1^18–20^. Correspondingly the site of the mutation is bound both by GATA1 and TAL1 in primary erythroblasts homozygous for the mutation^21^. In addition, we identified a strong binding motif for another key erythroid TF KLF1, in the immediate vicinity of the GATA1 motif (Fig. 2A). Using ChIP-seq we found a near two-fold increase in GATA1 occupancy and over four-fold increase in KLF1 binding in the vicinity of the C-SNV (Fig. 2B). This is of interest since both GATA1 and KLF1 are master regulators of erythroid genes^22–25^, have a role in establishing and/or maintaining chromatin conformation^26–29^, and are often found to co-occupy the same sites in erythroid cells^30^. Of interest, we analysed the predicted chromatin accessibility of the human α-globin gene cluster with and without the candidate SNV using a recently established, convoluted neural network (DeepHaem) [https://github.com/rschwess/deepHaem]. DeepHaem has previously been used to predict chromatin accessibility and its effect on higher order chromatin structures using DeepC^31^. This network predicts that the C-SNV mutation alone is sufficient to create an open chromatin site whose accessibility increases as cells differentiate along the erythroid pathway (Supplementary Fig. 2b).

To determine experimentally whether the T to C transition is the sole causative variant we introduced this point mutation homozygously into wild type cells and reverted the mutation in mutant cells (for genetic screening and quality control see Supplementary Fig. 2). Editing the mutant C allele to a T (C–T lines) abolished the accessible chromatin site surrounding the mutation and transcription at the site of the SNV became undetectable (Fig. 2C–E). Conversely, introducing the mutant C allele in wild type cells (T–C lines) produces a region of accessible chromatin at the site of the SNV and transcription of this region (Fig. 2C–E). We performed qPCR to determine if the T to C transition causes downregulation of α-globin. The C–T lines showed an over two-fold increase in α-globin mRNA when compared to its isogenic control C-SNV line. Conversely, the T–C lines showed over 55% reduction in α-globin transcript (Fig. 2D). These results prove that the T to C transition is both necessary and sufficient to cause the observed phenotype and that the accumulation of erythroid TFs around the TSS is causative rather than corelative in creating a new promoter element.

### The new promoter sequesters enhancers away from native promoters

**Fig. 3:**
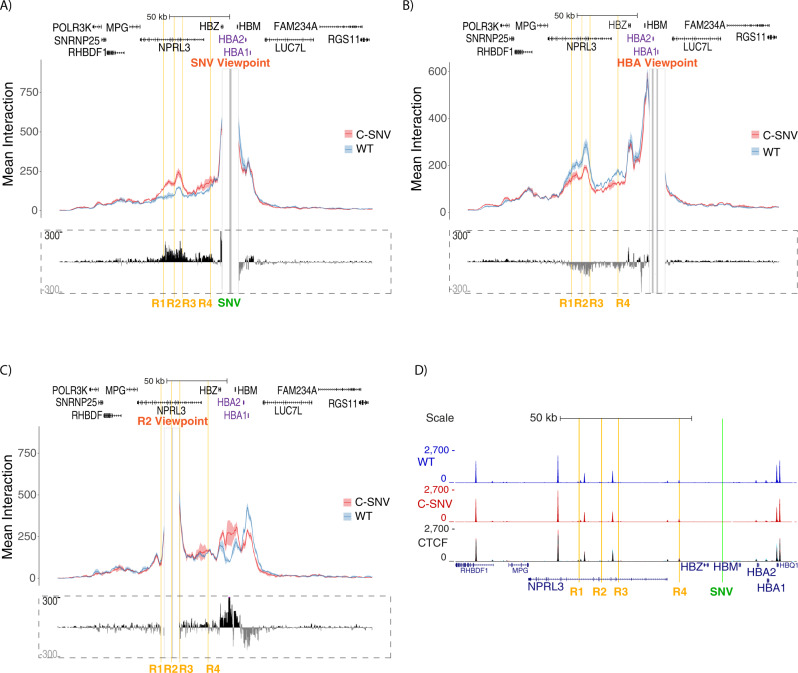
New promoter sequesters interactions with the α-globin enhancers away from their cognate promoters. **A** Top panel shows overlaid, normalised Capture-C data for the *novel* promoter (SNV Viewpoint) in erythroid cells derived from 3 independent differentiation experiments: 3 biologically independent samples of wild type iPSC line AH017-13 (WT in blue) or 3 clones of C-SNV iPSCs obtained from the same patient material (C-SNV in red). The mean, plus and minus one standard deviation (S.D.), of sliding 5 kb windows are visualised as a dark coloured line and a lighter coloured shadow, respectively. Differential tracks (in dashed rectangle) show a subtraction (C-SNV minus WT) of the mean value of meaningful interactions per restriction fragment: positive values (in black) display an increase in interaction over a region in the mutant cells (C-SNV) compared to wild type, while negative values (in grey) indicate a decrease. The enhancer elements (R1 to R4) are highlighted in orange, the site of the T to C mutation is highlighted in green (SNV), gene annotation by Refseq is in black (except α-globin genes in purple). Coordinates (hg19) chr16:69,200-327,999. **B** Data presented as in **A**, viewpoint is promoters of HBA1 and HBA2. **C** Data presented as in **A**, viewpoint is enhancer R2. **D** ChIP-seq for CTCF, enhancer elements (R1 to R4) are highlighted in orange, the site of the T to C mutation is highlighted in green (SNV), gene annotation by Refseq is in blue. Read-densities represent an average of 3 independent differentiation experiments, 3 replicates for wild type iPSC line AH017-13 (WT in blue) or 3 C-SNV iPSC clones (C-SNV in red) differentiated to erythroblasts. The third track (CTCF) displays WT and C-SNV overplayed, black indicates shared signal while red and blue indicate signal unique for the mutant and wild type lines, respectively. Coordinates (hg19) chr16:108,000-238,000.

The emergence of a new promoter, induced by key erythroid transcription factors like GATA1, in an erythroid-specific locus suggests that its expression may be regulated by the local enhancers. Indeed, GATA1 is thought to promote chromatin looping through its binding partners, among those that have shown to play a role in chromatin conformation are the co-factor FOG1^32–34^ and the multimeric LDB1-complex. The LDB1-complex consists of TAL1/E-protein heterodimer bound to GATA1 via LMO2 and LDB1^35^ and is thought to achieve transcriptional regulation through facilitating interactions between distal regulatory elements and promoters^23,36,37^. Genome wide studies have identified frequently occurring composite TAL1 and GATA1 binding sites known as Ebox-GATA1 motifs at erythroid regulatory elements^38^ confirming their co-association. In addition, GATA1 has been shown to cooperate with the TF NF-E2 to contribute to the recruitment of the ATPase component of SWI/SNF chromatin remodelling complex–BRG1, in order to facilitate the formation of accessible chromatin^38^ and to maintain higher order chromatin structure^27,29^. In particular, BRG1 is required for long range interactions in the mouse α-globin locus^39^ where GATA1 binds both the enhancers and the globin promoters early on during erythroid differentiation^40^. To test if the C-SNV element comes into close proximity with the α-globin enhancers, we assessed the interaction between key sites in the α-globin locus using Capture-C^41^. Analysis of the interaction profiles of C-SNV erythroid cells and wild type cells, from the viewpoint of the new promoter, shows an increase in mean interaction frequency between the α-globin enhancers and the new promoter (Fig. 3A). To determine if this interaction occurs at the expense of contacts between the α-globin enhancers and their cognate promoters we performed Capture-C from the viewpoint of the promoters of the α-globin genes (*HBA1* and *HBA2*). This showed a marked decrease in mean interaction frequency between the α-globin promoters and enhancers in the presence of the new C-SNV promoter (Fig. 3B). To confirm that this observation holds true from the point of view of the α-globin enhancers we also performed Capture-C from the R2 enhancer (Fig. 3C). This demonstrates that in erythroid cells homozygous for the C allele, the α-globin enhancers display a higher mean interaction frequency over the region of the active C-SNV promoter and a reduction of interactions with the α-globin promoters, and that this is likely to be an important component underlying the reduction in α-globin expression.

Reduced contacts between the enhancers and the α-globin promoters in the presence of the newly formed promoter could be explained by a model of mutually exclusive promoter competition, which has previously been proposed to explain several forms of complex gene regulation^42,43^. Alternatively, this could be explained by the formation of an insulator element by the new promoter, reminiscent of that observed at a subset of Drosophila promoters which act as enhancer blockers; a type of insulator element that restricts enhancer–promoter interactions^44^. Structural boundary elements that regulate chromatin architecture are commonly associated with the presence of the CCCTC-binding factor (CTCF)^45,46^. A CTFC ChIP-seq analysis showed that there are no new CTCF peaks that appear in the C-SNV lines, demonstrating that the activity of the C-SNV promoter alone is the cause of the shift in chromatin interactions (Fig. 3D).

### The competition model cannot explain the full effect of the new promoter

**Fig. 4:**
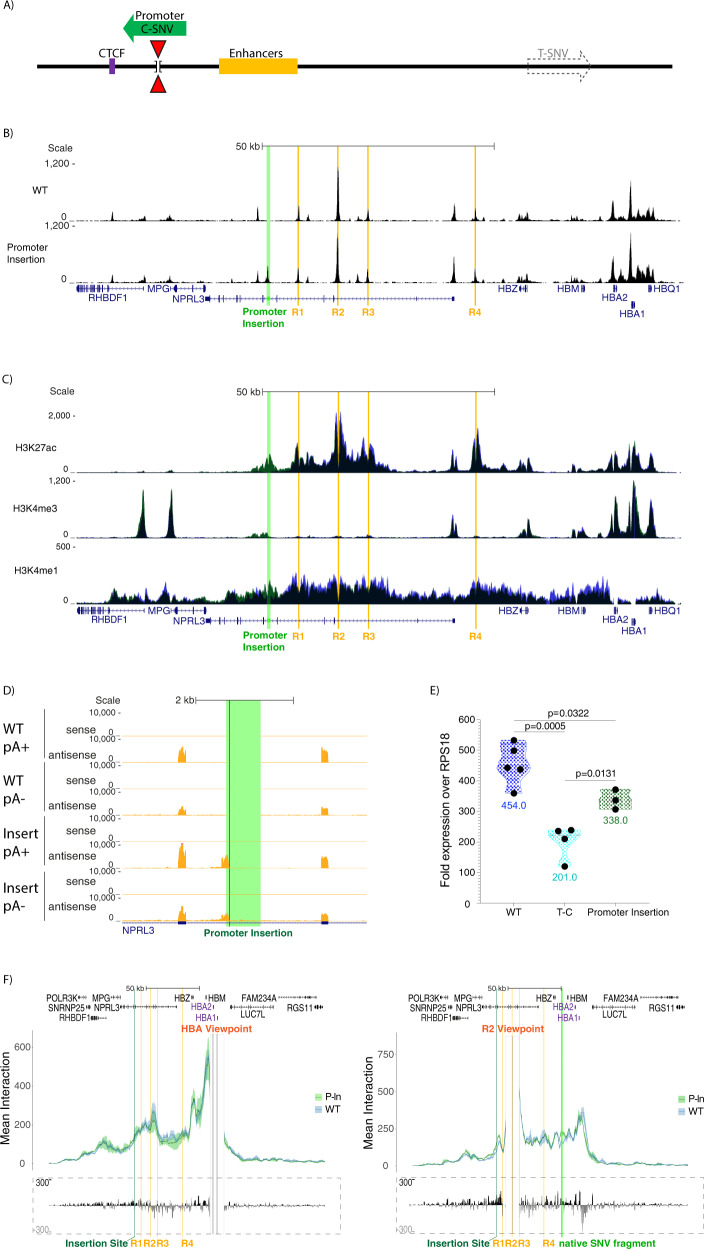
Placing the novel promoter behind the enhancers does not recapitulate the effect seen in the native position. **A** The active C-SNV promoter sequence (703 bp) was placed behind the α-globin enhancers before the CTCF site delimiting the chromatin interactions within the locus. **B** ATAC-seq: the enhancer elements (R1–R4) are highlighted in orange, the inserted C-SNV promoter is highlighted in green (Promoter insertion), gene annotation by Refseq is in blue. Read-densities represent an average of 3 independent differentiation experiments for wild type iPSC line SB-AD2-01 (labelled WT), 3 clones of edited SB-AD2-01 cells where the 703 bp of the active C-SNV promoter sequence is inserted behind R1 in the antisense orientation (labelled as Promoter Insertion). Reads from the Promoter Insertion cells were mapped to a custom genome which contains the 703 bp promoter insertion in antisense. Coordinates (hg19) chr16:108,000-238,000. **C** ChIP-seq, highlighted regions are as in a). Read-densities represent an average of 3 independent differentiation experiments for wild type iPSC line SB-AD2-01 (in blue), 3 clones of Promoter Insertion cells (in green). The tracks are overplayed, the darker colour indicates shared signal while green and blue indicate signal unique for the promoter insertion and wild type lines, respectively. Reads from the Promoter Insertion cells were mapped to a custom genome. Coordinates (hg19) chr16:108,000-238,000. **D** Strand-specific RNA-seq of polyadenylated selected (pA+) and non-polyadenylated (pA−) RNA, read density (in RPKM) represents an average of 3 independent differentiation experiments, 3 replicates for wild type line SB-AD2-01 (WT) or 3 clones of Promoter Insertion cells (Insert). The region of the promoter insertion is highlighted in green, the TSS is marked by a black line, gene annotation by Refseq is in blue. Reads from the Promoter Insertion cells were mapped to a custom genome. Coordinates (hg19) chr16:147,000-152,000. **E** qPCR quantification of *HBA1/HBA2* in reference to *RPS18* in mRNA obtained from independent differentiation experiments: 5 from wild type iPSC line SB-AD2-01 (WT), 4 clones of T–C cells (T–C), 3 clones of Promoter Insertion cells (Promoter Insertion). WT (*n* = 5) in blue, T–C (*n* = 4) in cyan, Promoter Insertion (*n* = 3) in green. Violin plots display median (dashed black line) quartile lines (coloured dotted line) and individual data points (black dots). *P* values are obtained using unpaired, two-tailed student *t-*test. **F** Top panel shows overlaid, normalised Capture-C data for either the promoters of the α-globin genes (left graph) or enhancer R2 (right graph) in erythroid cells derived from 3 independent differentiation experiments: 3 biologically independent samples of wild type iPSC line SB-AD2-01 (WT in blue) or 3 clones of Promoter Insertion cells (P-In in green). The mean, plus and minus one standard deviation (S.D.), of sliding 5 kb windows are visualised as a dark coloured line and a lighter coloured shadow, respectively. Differential tracks (in dashed rectangle) show a subtraction (P-In minus WT) of the mean value of meaningful interactions per restriction fragment. Reads were mapped to the wild type genome. Coordinates (hg19) chr16:69,200-327,999.

We next wanted to understand whether the effect of the C-SNV promoter was due to mutually exclusive promoter competition which should result in the promoter exerting its effect on chromatin interactions and gene expression irrespective of its position in the sub-TAD. Alternatively, if the C-SNV promoter is acting as an enhancer blocker its location (and perhaps orientation) relative to the native promoters and enhancers in the locus would determine its effect on gene regulation. To test the competition model, we placed the sequence of the active C-SNV promoter upstream of the α-globin enhancers in a wild type line, in the antisense orientation, directed away from the enhancers (Fig. 4A) (for genome editing design and genetic screen see Supplementary Fig. 3). Importantly, this insertion still lies within the α-globin sub-TAD and therefore should be equally accessible to the enhancers. In addition, the ectopically placed C-SNV promoter lies closer to the major R2 enhancer (14 kb) than the α-globin genes (59 kb) or the original position of the C-SNV (46 kb). ATAC-seq showed that the transposed C-SNV promoter sequence opens chromatin in this new position (Fig. 4B). The associated chromatin showed a large increase in H3K27ac and H3K4me3, and a less pronounced increase in H3K4me1 (Fig. 4C) together with unidirectional transcription originating at the TSS of the transposed C-SNV promoter (Fig. 4D). This transcript extends away from the enhancers on what is now the antisense strand of DNA. Thus, when placed within the sub-TAD upstream of the enhancers, the C-SNV sequence can still act as a *bona fide* promoter. Interestingly, when the promoter was placed in the sense orientation, it again created a region of open chromatin marked by H3K27ac and H3K4me1 but not by H3K4me3. In this case there were no detectable RNA transcripts (Supplementary Fig. 4). Together, these observations suggest that the orientation of this promoter relative to other elements may determine whether or not it is recognised as a promoter.

To test if the transposed transcriptionally active C-SNV promoter affected α-globin expression, we performed qPCR analysis. There was a reduction (25.6%, *p* = 0.0322) in mean α-globin transcript levels seen between cells where the transcriptionally active C-SNV promoter is placed upstream of the α-globin enhancers and wild type cells. However, we observed a larger reduction (55.7%, *p* = 0.0005) in cells of the same genomic background in which the C-SNV promoter is in its original position between the enhancers and α-globin promoters (T-C) (Fig. 4E). This is not sufficient to explain the severity of the phenotype that the C-SNV promoter produces in its native location. To determine if the transposed C-SNV promoter exerts any effect on chromatin interactions in the α-globin locus when placed upstream of the enhancers we performed Capture-C from enhancer R2 and the promoters of *HBA1* and *HBA2*. This showed no major change in chromatin interactions between the α-globin promoters and their enhancers in line with the minor change in expression (Fig. 4F. These observations indicate that placing the element within the sub-TAD but outside of the region between the enhancers and promoters does not significantly interfere with enhancer-promoter interactions as judged by 3 C experiments. This is in line with our recent studies showing that the extension of the α-globin domain to encompass additional genes does not result in promoter competition but results in the formation of a larger chromatin hub where the extra elements together with the native elements take part in multiway interactions without causing major changes in α-globin expression^47^. Together these findings suggest that although there may be some competition between the newly formed promoter and the enhancers, the C-SNV promoter may also act by blocking enhancer-promoter interactions when placed between the α-globin enhancers and their promoters. One caveat of this interpretation lies in the uncertainty of whether promoter strength might determine its ability to successfully compete with other promoters. Indeed, the signal levels of the promoter mark H3K4me3 are much lower in the promoter insertion lines, for this reason we returned to examining the promoter in its native position.

### The new promoter acts as orientation-dependent enhancer-blocker

**Fig. 5:**
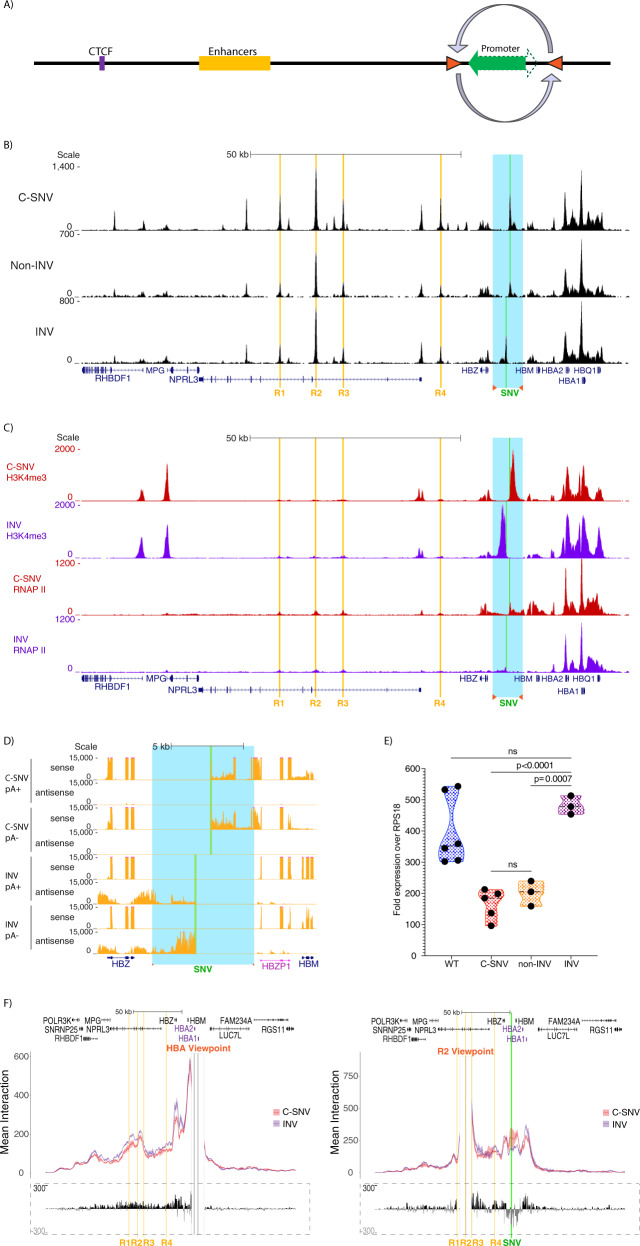
Changing the orientation of the promoter alleviates repression and increases α-globin enhancer-promoter contacts. **A** The native promoter was inverted using heterotypical loxP sites located 4011 bp upstream and 2975 bp downstream of the XCPE1 TSS (fragment chr16:205,734-212,720, hg19). **B** ATAC-seq: the enhancer elements (R1 to R4) are highlighted in orange, the site of the T to C mutation is highlighted in green (SNV), the inverted region is highlighted in light blue, gene annotation by Refseq is in blue. Read-densities represent an average of: 3 C-SNV iPSC clones (labelled C-SNV), 3 clones of edited C-SNV cell line LA01 where the heterotypical loxP sites are inserted but the C-SNV promoter segment is not inverted (labelled nonINV), 3 clones of edited patient line LA01 where the C-SNV promoter segment is inverted (labelled INV). Reads for C-SNV and nonINV were mapped to the normal genome, reads for INV were mapped to a custom genome where a 7 kb segment containing the C-SNV promoter is inverted. Coordinates (hg19) chr16:108,000-238,000. **C** ChIP-seq, highlighted regions are as in a). Read-densities represent an average of 2 or 3 independent differentiation experiments, 3 C-SNV iPSC clones (C-SNV in red), 2 clones of INV cells (INV in purple). Reads for C-SNV were mapped to the normal genome, reads for INV were mapped to a custom genome. Coordinates (hg19) chr16:108,000-238,000. **D** Strand-specific RNA-seq of polyadenylated selected (pA+) and non-polyadenylated (pA-) RNA, read density (in RPKM) represents an average of 2 or 3 independent differentiation experiments: 3 C-SNV iPSC clones (C-SNV) or 2 clones of INV cells (INV). The inverted segment is highlighted in blue, the location of the LoxP sites is marked by orange triangles, the region of the T to C mutation is highlighted in green (SNV), gene annotation by Refseq is in blue with pseudogenes in pink. Reads for C-SNV were mapped to the normal genome, reads for INV were mapped to a custom genome. Coordinates (hg19) chr16:202,000-217,000. **E** qPCR quantification of HBA1/HBA2 in reference to RPS18 in mRNA obtained from independent differentiation experiments: 3 independent wild type iPSC lines differentiated twice (WT), 3 C-SNV iPSC clones (C-SNV) differentiated twice (one replicate removed as an outlier), 3 clones of nonINV cells (nonINV), 3 clones of INV cells (INV). WT (*n* = 6) in blue, C-SNV (*n* = 5) in red, nonINV (*n* = 3) in orange, INV (*n* = 3) in purple. Violin plots display median (dashed black line) quartile lines (coloured dotted line) and individual data points (black dots). P-values are obtained using unpaired, two-tailed student *t*-test. **F** Top panel shows overlaid, normalised Capture-C data for either the promoters of the α-globin genes (left graph) or enhancer R2 (right graph) in erythroid cells derived from 3 independent differentiation experiments: 3 C-SNV clones (C-SNV in red) or 3 clones of INV cells (INV in purple). The mean, plus and minus one standard deviation (S.D.), of sliding 5 kb windows are visualised as a dark coloured line and a lighter coloured shadow, respectively. Differential tracks (in dashed rectangle) show a subtraction (INV minus C-SNV) of the mean value of meaningful interactions per restriction fragment. Reads were mapped to the wild type genome. Coordinates (hg19) chr16:69,200-327,999.

Elements which limit the interactions between promoter and enhancer in mammalian cells such as boundaries have been shown to commonly act in an orientation dependent manner^48^. It is possible that this novel element may act in a similar manner, although we could find no evidence for orientation-specificity in promoters that act as enhancer-blockers in Drosophila or yeast. To test if the orientation of the C-SNV element contributes to its ability to block enhancer-promoter interactions, we inverted the active C-SNV promoter sequence in its natural position (Fig. 5A) (for genome editing design and genetic screen see Supplementary Fig. 5). Asking if the element still acted as a unidirectional promoter after the inversion we showed that the inverted element is still capable of opening chromatin, is marked by H3K4me3 and is capable of recruiting RNAP II (Fig. 5B, C). Importantly, the ATACseq and H3K4me3 signal levels over the C-SNV promoter were similar to those in the C-SNV lines, suggesting that even when inverted promoter strength is maintained. We also proved that the unidirectional nature of the transcriptional activity is preserved, with transcripts with similar levels of expression now extending towards the enhancers (Fig. 5D). To determine whether the C-SNV promoter still blocked enhancer-promoter interactions when inverted, we performed Capture-C from the α-globin promoters and enhancer R2, and qPCR analysis for α-globin expression. This showed that after inversion of the C-SNV promoter the α-globin promoters interact more frequently with their enhancers while the contacts between the α-globin enhancers and the inverted C-SNV promoter decrease (Fig. 5F). Consistent with this, the levels of α-globin mRNA return to wild type levels following the inversion of the C-SNV promoter (Fig. 5E). Since the C-SNV sequence appears to act as an equally strong promoter in either orientation, it should “compete” equally for the enhancer in either orientation. By contrast, we have shown that the inverted C-SNV promoter no longer causes local gene mis-regulation. These observations support the hypothesis that the new (C-SNV) promoter acts as an enhancer-blocker and that this effect is constrained by its orientation in the active locus.

## Discussion

**Fig. 6:**
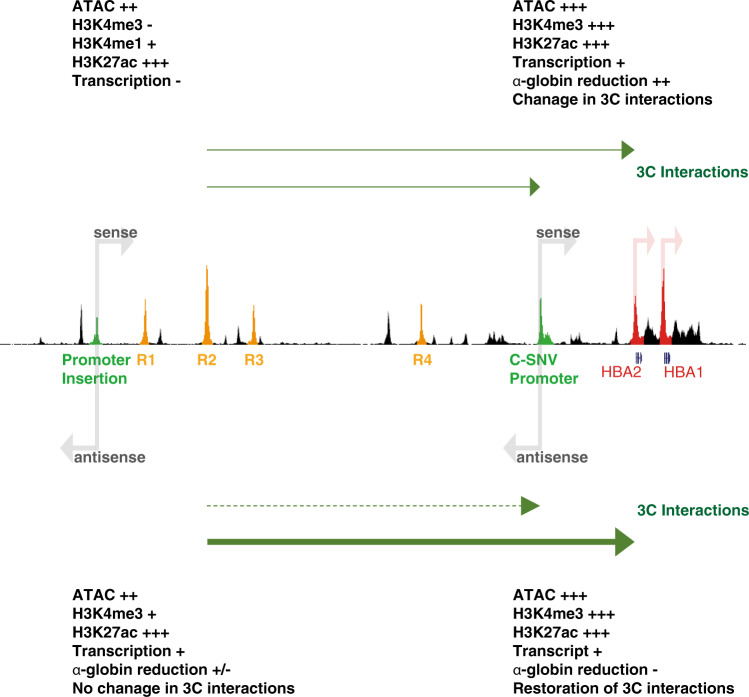
C-SNV promoter behaviour in different contexts within the α-globin locus. Schematic shows the effect the active C-SNV promoter has on the regulation of the α-globin locus when placed either in its native location (labelled C-SNV promoter in green) or inserted behind the α-globin enhancers (labelled Promoter insertion in green). Elements of importance are highlighted on an artificially merged ATACseq track: enhancers R1-R4 in orange and *HBA2/1* genes in red. The effects of the different genomic arrangements are adjacent to the conformation of the element on the schematic.

In this work, we have found that a gain of function SNV causes the emergence of an active promoter in a tissue-specific gene locus, disrupting normal chromatin interactions and gene regulation. By changing the position and orientation of the novel promoter, we show that rather than simply competing for the activity of the enhancer within a defined sub-TAD, the new promoter acts in an orientation-dependent manner. It predominantly disrupts chromatin interactions only when placed between the enhancers and the promoters of the α-globin genes, and only in one orientation (see Fig. 6). This raises two possibilities to explain the effects of the C-SNV promoter at its natural location between the enhancers and the α-globin promoters. One is that the resulting reduction in α-globin expression could reflect a role for the C-SNV element as an orientation-dependent enhancer-blocker. Alternatively, it could be that the enhancer-promoter interaction is weaker when the C-SNV promoter is inverted with respect to the enhancer, with the promoter inversion altering the enhancer’s ability to recognise its target. This, in turn, might reduce the C-SNV promoter’s ability to compete for the enhancer activity. However, there is no current evidence, to our knowledge, to suggest that enhancers differ in their interactions with respect to the orientation of their cognate promoters. If so, this would contest the commonly held hypothesis that enhancers and promoters interact equally regardless of their orientation^49^. Of interest, we have also recently shown that the α-globin promoters themselves may act to delimit enhancer contacts within the context of the sub-TAD^50^. Together these observations support the concept that promoters may act to block enhancer-promoter interactions in mammals as in Drosophila. However, the mechanism(s) by which a promoter may act as an orientation dependent enhancer-blocker are not yet known. We can only speculate that the motif grammar, the conformation of multiprotein complexes at the promoter and/or the direction and the act of transcription may all play a role in this. Further studies varying these parameters will be required to determine such mechanisms. The observation that a tissue-specific promoter can act to block an enhancer promoter interaction in an orientation-dependent manner raises the question of whether other promoters, most notably those that are situated at the edges of self-interacting domains, might exhibit the same type of effect and whether their orientation might serve as an additional mechanism that cells use to direct specificity of interactions and shape local chromatin conformation. Finally, variants like C-SNV which generate de novo regulatory elements can only be identified by observation in the correct genotype or, as shown here, through predictive machine learning approaches. These findings should prompt others to re-evaluate SNVs lying outside of known regulatory elements when studying human traits associated with natural variation.

## Methods

### Induced pluripotent stem cell maintenance

Human iPSCs were cultured at 37 °C 5% CO_2_ in mTeSR1 medium (StemCell Technologies) on tissue culture treated plastic ware (Corning) coated with Human ESC Qualified Matrigel (Corning) with daily medium changes. Cultures were passaged 1 in 8 when 70–80% confluent using a non-enzymatic method employing a 0.02% EDTA containing solution (Versene from Lonza). Frozen stocks were stored in 50% mTeSR1 30% Knock-Out Serum Replacement (Gibco) 10% Knock-Out DMEM (Life Technologies) 10% DMSO (SIGMA) under liquid Nitrogen vapour. Frozen stocks were resurrected in mTeSR1 medium containing 10 μM ROCK inhibitor (RnD).

Wild type human induced pluripotent stem cell lines SB-AD2-01, SB-AD3-01 and AH017-13 were obtained from Oxford StemBANCC. C-SNV human induced pluripotent stem cell lines LA01, LA06 and LA13 were generated and validated in house. Ethical approval for the study was granted by North West Research Ethics Committee of NHS National Research Ethics Services, Ref: MREC 03/8/097. The experimental procedures in this study were carried out in accordance with the approved guidelines. Informed consent was obtained for the generation of human derived iPSC lines.

### Reprogramming to pluripotency

Reprogramming EBV immortalised B-cells from an individual homozygous the C allele (C-SNV) was performed using the CytoTune-iPS 2.0 Sendai Reprogramming Kit (Life Technologies) according to manufacturer’s protocol using the feeder-based reprogramming method on Mitomycin C inactivated MEF plated dishes. When iPSC colonies were ready for transfer (day 18–20) they were transferred onto a Matrigel coated plate in mTeSR1 medium. Subsequently, the lines were maintained for 2 months until they reached passage 17–20 and then quality control was performed, and the lines were stored in cryogenic store. Human iPSC lines at passage 17–20 were assessed for pluripotency marker expression using the PSC 4-Marker Immunocytochemistry Kit (Invitrogen) as per the manufacturer’s protocol. Copy number variation analysis was performed on high quality genomic DNA using the Infinium HD assay on Human CytoSNP 12 Beadchip v2.1 (Illumina) at the Wellcome Trust Centre for Human Genetics (Oxford). Data was analysed using the KaryoStudio software package (Illumina).

### Genome editing of iPSCs

DNA was introduced into human iPSCs by a lipofection based method using Fugene 6 (Promega). Up to 8 μg of DNA was used per 10^6^ cells, equal amounts of single-stranded repair template DNA and pSpCas9(BB)-2A-Puro (PX459) V2.0 plasmid DNA. For point mutation and lox site insertion single stranded oligonucleotides were ordered from IDT. For promoter insertion single stranded DNA was generated in house using λ exonuclease and a 5’ phosphorylated primer (for sequences see Supplementary Table 1). Exogenous DNA containing cells were selected using puromycin for 24 h, cells were then maintained as normal for one passage. The puromycin resistant cells were brought to a single cell suspension using Accutase (Millipore) and seeded at low density (~800 cells in suspension per 10cm^2^). After 7–9 days iPSC colonies were picked into a 96-well plate coated with Matrigel in mTeSR1 medium with 10 μM ROCK inhibitor. The 96 well plate was split to create 2 replica plates, one of the replica plates was used for genotyping the other one was used to expand the clones with correct edits.

### Erythroid differentiation of iPSCs

Prior to the initiation of differentiation protocol, human iPSCs were cultured for at least 15 days on Vitronectin (Invitrogen) coated plates (Corning; TC treated) in STEMPRO hESC media (Invitrogen) supplemented with 20 ng/ml bFGF (RnD). Cultures were maintained in this way for 5–6 passages at high confluency and split 1 in 4 when over 85% confluent. Human iPSCs were differentiated as published by Olivier et al.^12^ and harvested on day 21 of the differentiation protocol. Flow cytometry analysis was performed on 2 × 10^5^ cells resuspended in 200 μl of 2% bovine serum albumin (SIGMA) in phosphate buffered saline and labelled for 20 min at 4 °C with 1:200 dilution of fluorescein isothiocyanate (FITC) conjugated anti-CD71 (BD Biosciences; 555536) and 1:200 dilution of phycoerythrin (PE) conjugated anti-CD235a (BD Biosciences; 340947). After staining cells were harvested and resuspended in PBS containing 0.02% Hoechst 33258 pentahydrate nucleic acid stain (Invitrogen). Analysis was performed on an Attune NxT Flow Cytometer (Invitrogen) and subsequently using FlowJo software for gating on viable single cell events and representing the data as a dot plot. Morphological analysis was preformed using a modified Wright stain on a Hemateck 03564647 slide strainer (BAYER). The slides were then imaged on a Nikon inverted microscope.

### RNA expression analysis and TSS mapping

Total RNA was isolated from 5 × 10^6^ day 21 erythroid cells using the Direct-zol RNA kit (Zymo) as per manufacturer’s instructions. DNase I digestion was performed on column. RNA purity was assessed using Nanodrop ND-1000 spectrophotometer (ThermoFisher Scientific). RNA integrity was analysed using RNA ScreenTape Assay (Agilent). For qRT-PCR 500 ng of DNA free RNA was reverse-transcribed using the Superscript III First Strand Synthesis kit (Life Technologies) and the cDNA solution was treated with RNase H according to manufacturer’s protocol. Commercially validated TaqMan assays (Applied Biosystems) were used in qRT-PCR experiments, these can be found in Supplementary Table 1. Custom TaqMan assays were used for detecting the novel transcript produced from the C-SNV promoter (sequences for these are found in Supplementary Table 1). Reactions were carried out in triplicate in 96-well plates on the StepOnePlus Real-Time PCR System (ThermoFisher Scientific). Quantification of data was made using the ΔCt method to determine the relative abundance of the gene of interest compared to a housekeeping control *RPS18*. Statistical significance was determined using unpaired, two-tailed student t-test. For RNA-seq libraries, 1 μg of total RNA was depleted of rRNA using the rRNA Removal Kit (Illumina) according to the manufacturer’s instructions. NEBNext Poly(A) mRNA Magnetic Isolation Module (NEB) was used to separate polyadenylated from non-polyadenylated RNA, subsequently strand-specific cDNA was synthesised, and the resulting libraries prepared for Illumina sequencing using the NEBNext Ultra II Directional RNA Library Prep Kit for Illumina (NEB) following the manufacturer’s instructions. Polyadenylated and non-polyadenylated RNA-seq libraries were sequenced on the NextSeq platform using High-output 75cycle kits (Illumina), paired end sequencing. Reads were aligned to the hg19 human genome (or custom genomes based on hg19 with corresponding edited sequence changes) build using the STAR aligner tool (version 2.7.4) and normalised to Reads Per Kilobase per Million (RPKM). The C-SNV promoter TSS was mapped using the FirstChoice RLM-RACE Kit (Invitrogen) with total RNA from day 21 erythroid cells from C-SNV line LA01 following manufacturer’s instructions (for 5’ RACE primer see Supplementary Table 1). The resulting PCR products were prepared for Illumina sequencing using the NEBNext Ultra II Directional RNA Library Prep Kit for Illumina (NEB) following the manufacturer’s instructions. Libraries were sequenced on the NextSeq platform using High-output 75cycle kits (Illumina), paired end sequencing. Reads were aligned to the hg19 human genome build using the bowtie 2 aligner tool (version 2.3.2 http://bowtiebio.sourceforge.net/bowtie2/index.shtml) and normalised to Reads Per Kilobase per Million (RPKM).

### ChIP-seq

Cells were fixed at 2.5 × 10^6^ cells/ml of 10% FCS RPMI media (Gibco) with 1% formaldehyde (SIGMA) for 10 min at RT. Fixed cells were lysed at 5 × 10^7^ cells/ml of cell lysis buffer (5 mM PIPES (Gibco), 85 mM KCl, 0.5% NP-40 (SIGMA)) + proteinase inhibitor (PI) (ROCHE) on ice for 20 min. The lysed fixed nuclei were lysed in nuclear lysis buffer (50 mM Tris-HCl, 10 mM EDTA (both from Life Technologies) 1% SDS (SIGMA)) + PI at a concentration of 1 × 10^8^ cells/ml. The nuclear lysis was sonicated using the Covaris S220 Focused-ultrasonicator in 130 μl microTUBEs (Covaris). The sonicated lysate was spun at max speed at 10 °C to remove insoluble material. The clear fixed chromatin was diluted 1 in 10 with RIPA buffer w/o SDS + PI (10 mM Tris-HCl, 1 mM EDTA, 0.5 mM EGTA (SIGMA), 1% Triton X-100 (SIGMA), 0.1% Na Deoxycholate (SIGMA), 140 mM NaCl). The diluted chromatin was precleared twice with 10 µl of a 1:1 mix of Protein A and Protein G Dynabeads (Invitrogen) by incubating at 4 °C for 30 mins and discarding the beads. The pre-cleared chromatin was incubated with the appropriate amount of antibody coupled to a 100 µl 1:1 mix of Protein A and Protein G Dynabeads at 4 °C overnight. Chromatin bound beads were washed twice with RIPA (10 mM Tris-HCl, 1 mM EDTA, 0.5 mM EGTA, 1% Triton X-100, 0.1% SDS, 0.1% Na Deoxycholate, 140 mM NaCl) +PI, then twice with high salt RIPA (same as RIPA but with 500 mM NaCl) +PI, once with LiCl RIPA (same as RIPA but with 250 mM LiCl instead of NaCl) +PI and twice with TE buffer. Chromatin was eluted off beads using 20 mM Tris-HCl, 5 mM EDTA, 50 mM NaCl, 1% SDS, 130 µg/ml RNase A, 65 µg/ml Proteinase K. Eluted chromatin and Input samples were incubated at 65 °C overnight to de-crosslink DNA from protein. DNA was isolated by Phenol-Cholorform (SIGMA) extraction as per manufacturer’s protocol. The ChIP library preparation was performed using the NEBNext Ultra DNA Library Preparation Kit for Illumina (NEB) as per manufacturer’s instructions. The libraries were sequenced on the NextSeq platform using High-output 75cycle kits (Illumina), paired end sequencing. Data were analysed using an in-house pipeline^51^. Versions of software packages used for the analysis include FASTQC 0.11.9, Bowtie 2.3.2, Samtools 0.1.19, Bedtools 2.25.0, Deeptools 2.2.2. Briefly, reads were mapped onto human genome build hg19 or custom genome builds based on human genome build hg19 using bowtie 2 (version 2.3.2 http://bowtiebio.sourceforge.net/bowtie2/index.shtml). PCR duplicates and ploidy regions were removed, and biological replicates were normalised to reads per 10^8^.

The antibodies used are: RNA Polymerase II N-20 antibody SC-899 (Santa Cruz Biotechnology) 6 μg per 1 ml of chromatin from 1 × 10^7^ cells; Monoclonal anti-acetyl-Histone H3 (Lys27) antibody 17-683 (Merck Millipore) 2 μg per 1 ml of chromatin from 1 × 10^7^ cells; Polyclonal anti-trimethyl-Histone H3 (Lys4) antibody 07-473 (Merck Millipore) 1 μg per 1 ml of chromatin from 1 × 10^7^ cells; Polyclonal anti-monomethyl-Histone H3 (Lys4) antibody ab195391 (Abcam) 4 μg per 1 ml of chromatin from 1 × 10^7^ cells; Polyclonal anti-GATA1 antibody ab11852 (Abcam) 8 μg per 1 ml of chromatin from 1 × 10^7^ cells; Polyclonal anti-CTCF antibody 07-729 (Merck Millipore) 10 μl serum per 1 ml of chromatin from 1 × 10^7^ cells. Polyclonal anti-KLF1 antibody was kindly provided by the Perkins Laboratory, Translational Research Institute, Brisbane, Australia (used 30 μl serum per 1 ml of chromatin from 1 × 10^7^ cells).

### ATAC-Seq

ATAC-seq was performed as previously published^52^. Briefly, 7 × 10^4^ cells per replicate were lysed and nuclei were isolated prior to tagmentation with Tn5 transposase (Illumina) for 30 min at 37 °C. Tagmented DNA was purified using the MinElute kit (Qiagen) and amplified using the NEBNext 2x Mastermix (NEB) and custom barcoded primers. The libraries were sequenced on the NextSeq platform using High-output 75cycle kits, paired end sequencing. Data were analysed using an in-house pipeline^53^. Briefly, reads were mapped onto human genome build hg19 or custom genome builds based on human genome build hg19 using bowtie 2 (version 2.3.2 http://bowtiebio.sourceforge.net/bowtie2/index.shtml). PCR duplicates and ploidy regions were removed, and biological replicates were normalised to reads per 10^8^. Mitochondrial DNA was excluded from the normalisation. Predicted open chromatin scores for reference and C-SNV sequences were generated using deepHaem^31^ using both variant alleles with 1 kb of flanking reference sequence.

### Next generation capture C

Next generation capture C was performed as previously published^41^. Briefly, 1 × 10^7^ cells per replicate were fixed in 2% formaldehyde. 3 C libraries were prepared following digestion with *NlaIII* enzyme in CutSmart buffer (NEB). Libraries for capture were generated using the NEBNext DNA library Prep Reagent Set (NEB) following the manufacturer’s protocol up to the addition of adapters then the libraries were indexed using the Herculase II Fusion Polymerase kit (Agilent) and the NEBNext Multiplex Oligos for Illumina Primers (NEB) following the manufacturer’s protocol. Capture was performed on pooled indexed libraries using biotinylated DNA probes (Supplementary Table 1) and the NimbleGen SeqCap EZ Reagent kit (Roche) following manufacturers protocol. The libraries were sequenced on the NextSeq platform using Mid-output 300 cycle kits, paired end sequencing. NG Capture-C data were analysed using the CaptureCompendium toolkit^53^ with human refence genome build hg18. Reporter counts were normalised to 10^5^ for the calculation of the mean and standard deviation for each replicate (*n* = 3). Mean reporter counts were divided into 250 bp bins and smoothed using a 5 kb window.

### Reporting summary

Further information on research design is available in the Nature Research Reporting Summary linked to this article.

## Supplementary information

Supplementary Information

Reporting Summary

## Data Availability

The data that support this study are available from the corresponding authors upon reasonable request. ATAC-seq, ChIP-Seq, RNA-seq and NG Capture-C raw data and bigwig files generated in this study are available under Gene Expression Omnibus (GEO) accession GSE159875 Analyses and coordinates referenced here are for either the hg19, hg18 human reference genomes, or custom genomes hg19_INV (inverted C-SNV promoter), hg19_Vas (promoter insertion in anti-sense behind enhancers) or hg19_Vs (promoter insertion in sense behind enhancers) based on reference human genome hg19, as indicated in figure legends. Sequences for chromosome 16 from the custom genomes are available in FASTA format as supplementary files in GEO Subseries GSE159871. Figures that have associated raw data files: Fig. 1A–C); Fig. 2C); Fig. 3A–D); Fig. 4B–F); Supplementary Fig. 4b–d). Source data are provided with this paper.

## Source data

Source Data
