# Supplementary material for: A gain-of-function single nucleotide variant creates a new promoter which acts as an orientation-dependent enhancer-blocker
Source: Nat Commun. 2021 Jun 21;12:3806. doi: 10.1038/s41467-021-23980-6 (PMC8217497; doi:10.1038/s41467-021-23980-6)
Supplement: Supplementary file 1 — Supplementary Information [file 41467_2021_23980_MOESM1_ESM.pdf]

A)

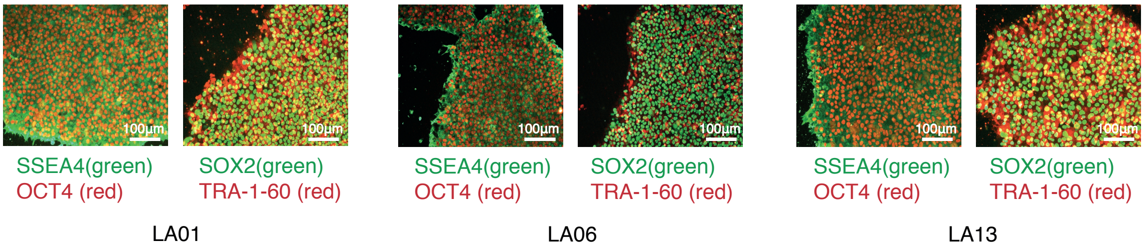

B)

Lines	Chr	Cytoband	Start	Stop	Length (bp)	Type	Genes affected
LA01, LA06, LA13	1	q31.1 q31.2 q31.3	189,752,408	198,117,642	8,365,234	LOH	FAM5C; RGS18; RGS21; RGS1; RGS13; RGS2; UCHL5; TROVE2; TROVE2; GLRX2; GLRX2; CDC73; MIR1278; B3GALT2; KCNT2; CFH; CFHR3; CFHR1; CFHR4; CFHR2; CFHR5; F13B; ASPM; ZBTB41; CRB1; DENND1B; DENND1B; C1orf53; LHX9; LHX9;
	11	p11.2 p11.12	45,173,290	51,530,241	6,356,951	LOH	PRDM11; SYT13; CHST1; DKFZp779M0652; SLC35C1; SLC35C1; CRY2; CRY2; MAPK8IP1; C11orf94; PEX16; GYLTL1B; PHF21A; CREB3L1; DGKZ; DGKZ; DGKZ; DGKZ; MDK; MDK; MDK; CHRM4; AMBRA1; HARBI1; KIAA0652; ARHGAP1; ZNF408; F2; CKAP5; SNORD67; LRP4; C11orf49; ARFGAP2; PACSIN3; DDB2; ACP2; ACP2; NR1H3; NR1H3; MADD; MADD; MADD; MYBPC3; SPI1; SLC39A13; PSMC3; RAPSN; CUGBP1; PTPMT1; KBTBD4; NDUFS3; FAM180B; C1QTNF4; MTCH2; AGBL2; FNBP4; NUP160; PTPRJ; OR4B1; OR4X2; OR4X1; OR4S1; OR4C3; OR4C45; OR4A47; FOLH1; LOC440040; OR4C13; OR4C12; LOC441601; LOC646813; OR4A5; OR4C46;
	X	p11.22 p11.21 p11.1	54,774,832	58,339,545	3,564,713	LOH	ITIH5L; MAGED2; MAGED2; MAGED2; SNORA11; TRO; PFKFB1; APEX2; ALAS2; PAGE2B; PAGE2; FAM104B; FAM104B; PAGE5; PAGE5; PAGE3; MAGEH1; USP51; FOXR2; RRAGB; KLF8; KLF8; UBQLN2; DKFZp686L07201; LOC442454; SPIN3; SPIN3; SPIN2B; SPIN2A; FAAH2; ZXDB; ZXDA;

C)

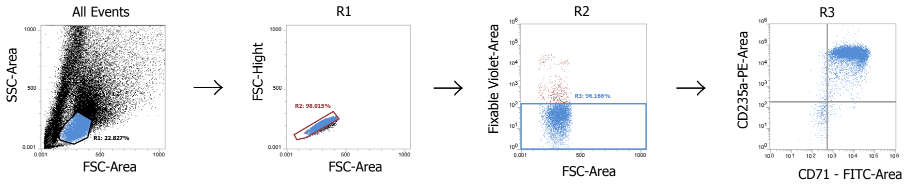

D)

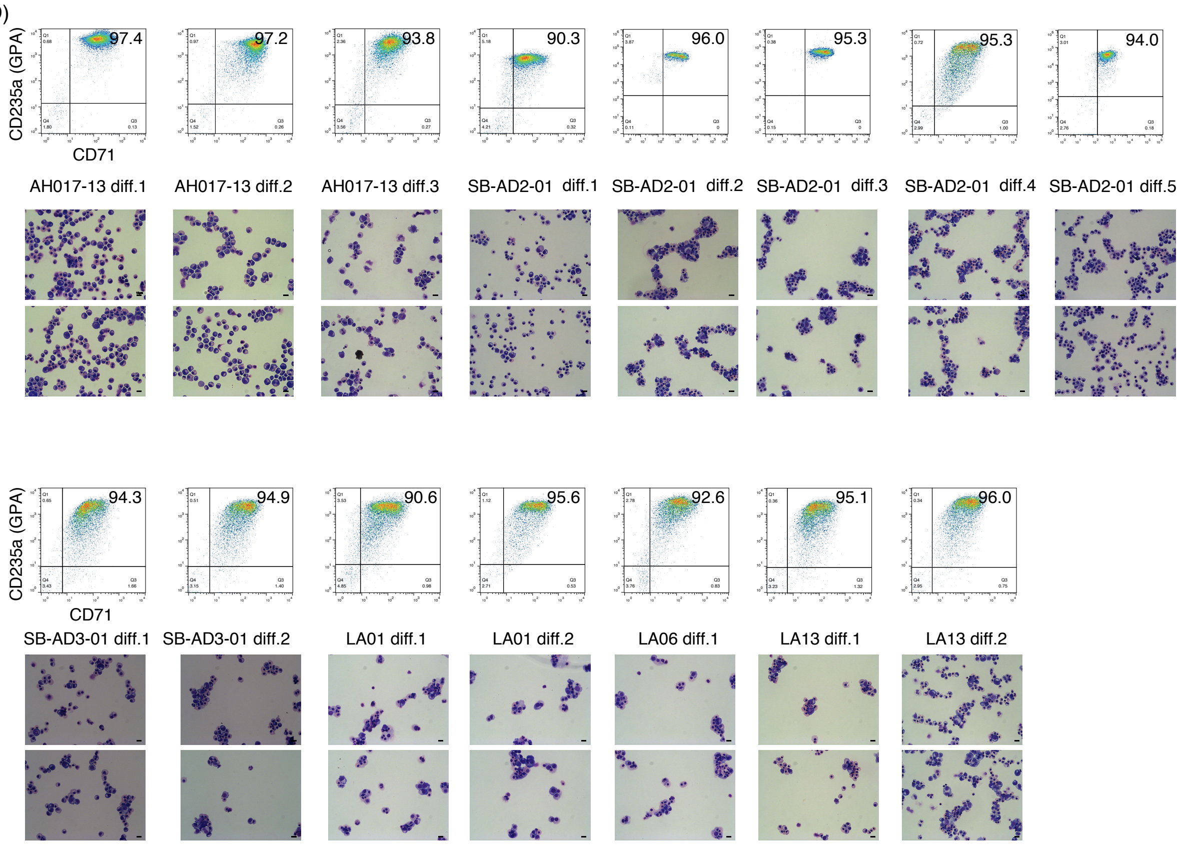

Supplementary Figure 1. C-SNV iPSC line quality control and differentiation characterisation

A) Pluripotency marker immunofluorescence for C-SNV iPSC lines LA01, LA06 and LA13. Experiment was performed once; representative images are displayed. The first column shows overlaid staining for SSEA4 in green and OCT4 in red, the second column shows overlaid staining for SOX2 in green and Tra-1-60 in red. **B)** Table showing the copy number variation found in iPSC lines LA01, LA06 and LA13 using the HumanCytoSNP-12 array from Illumina. The first column contains the name of the line; the second is the chromosome in which the CNV is found; the third column is the cytoband/s which the CNV

encompasses; the fourth and fifth column are the start and stop positions of the CNV region in human genome hg18; the sixth column indicates the type of mutation: LOH – loss of heterozygosity; Duplication - gain of one copy; 1 copy deletion - loss of one copy; the last column lists the genes affected by the abnormality. **C)** Gating strategy for FACS analysis. **D)** First panel: representative FACS analysis for cell surface iron Transferrin receptor - 1 (CD71) and cell surface glycoprotein Glycophorin A (CD235a or GPA) on day 21 of differentiation. Second panel: modified Wright stain of differentiation culture cells at day 21. Black bar represents 10µm. Each line was differentiated least twice. Line names are shown in black; instance of replicate is indicated after name.

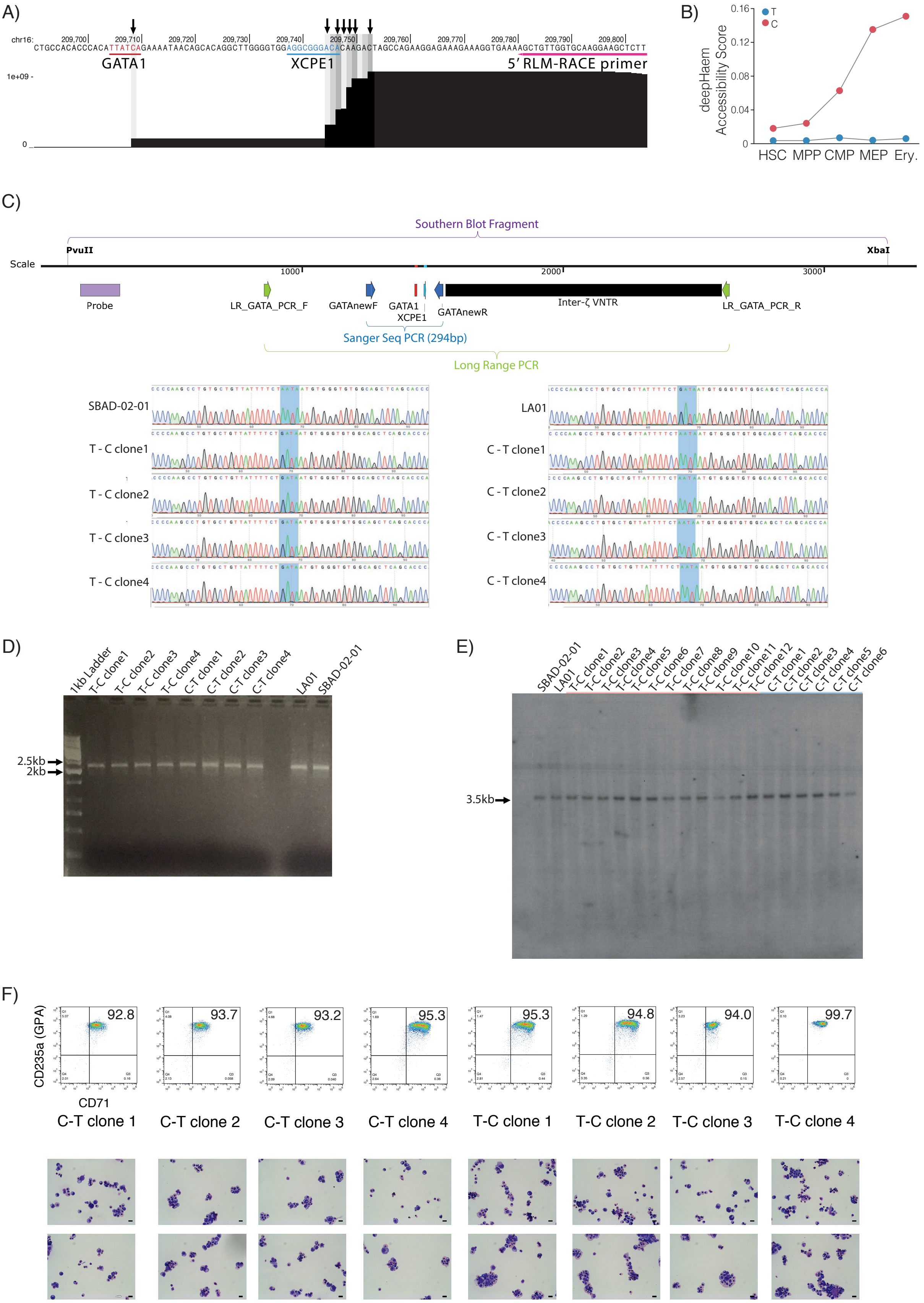

Supplementary Figure 2. Transcription start site mapping, variant editing quality control and differentiation characterisation

A) Transcription start site mapping as measured by 5' RLM-RACE coupled with NGS (RPKM normalised). The GATA1 site is indicated in red, the promoter element XCPE1 is indicated in blue, the 5' RLM-RACE primer is shown in pink and the 5' ends of reads are highlighted and indicated with black arrows. Read-densities represent one 5' RLM-RACE experiment from one erythroid differentiation of iPSC line LA01. Reads were mapped to the wild type genome. Coordinates (hg19) chr16: 209,691-209,804. **B)** DNA accessibility prediction scores, between 0 (closed) and 1 (open), using the deepHaem convolutional neural network for 1kb reference sequence (hg19; chr16:209,209-210,209) with each of the two alleles at chr16:209,709. Predictions were generated for Haematopoietic stem and progenitor cells (HSC), multi-potent progenitor cells (MPP), common myeloid progenitors (CMP), myeloid-erythroid progenitors (MEP) and CD71^{+} CD235^{+} Erythroid cells (Ery.). **C)** Top panel illustrates a 3.4kb fragment around the C-SNV (hg19, chr16:208,272-211,622). The Sanger sequencing PCR fragment (294bp) and associated primers are indicated in blue. The long-range PCR fragment (around 2.3kb: exact size undetermined as the region encompasses a VNTR) and associated primers are indicated in green. The Southern plot fragment (fragment should be >3.8kb based on long range PCR) and probe are highlighted in purple. Lower panel shows Sanger sequencing traces (sequencing primer GATAnewF): left – SBAD-02-01 unedited line and edited (T-C) clones; right – LA01 unedited line and edited (C-T) clones. **D)** Long range PCR (primers: LR_GATA_PCR_F + R; ladder: O'GeneRuler 1kb) for edited (T-C) clones and isogenic control line SBAD-02-01, and edited (C-T) clones and isogenic control line LA01. PCR was replicated twice; representative image is shown. **E)** Southern blot for edited (T-C) clones and isogenic control line SBAD-02-01, and edited (C-T) clones and isogenic control line LA01, digested with *PvuII* and *XbaI*, probe sequence can be found in *Supp.Tbl.1*. Southern blot was performed once. **F)** First panel: representative FACS analysis for cell surface iron Transferrin receptor - 1 (CD71) and cell surface glycoprotein Glycophorin A (CD235a or GPA) on day 21 of differentiation. Second panel: modified Wright stain of differentiation culture cells at day 21. Black bar represents 10µm. Each clone was differentiated once. Line names are shown in black.

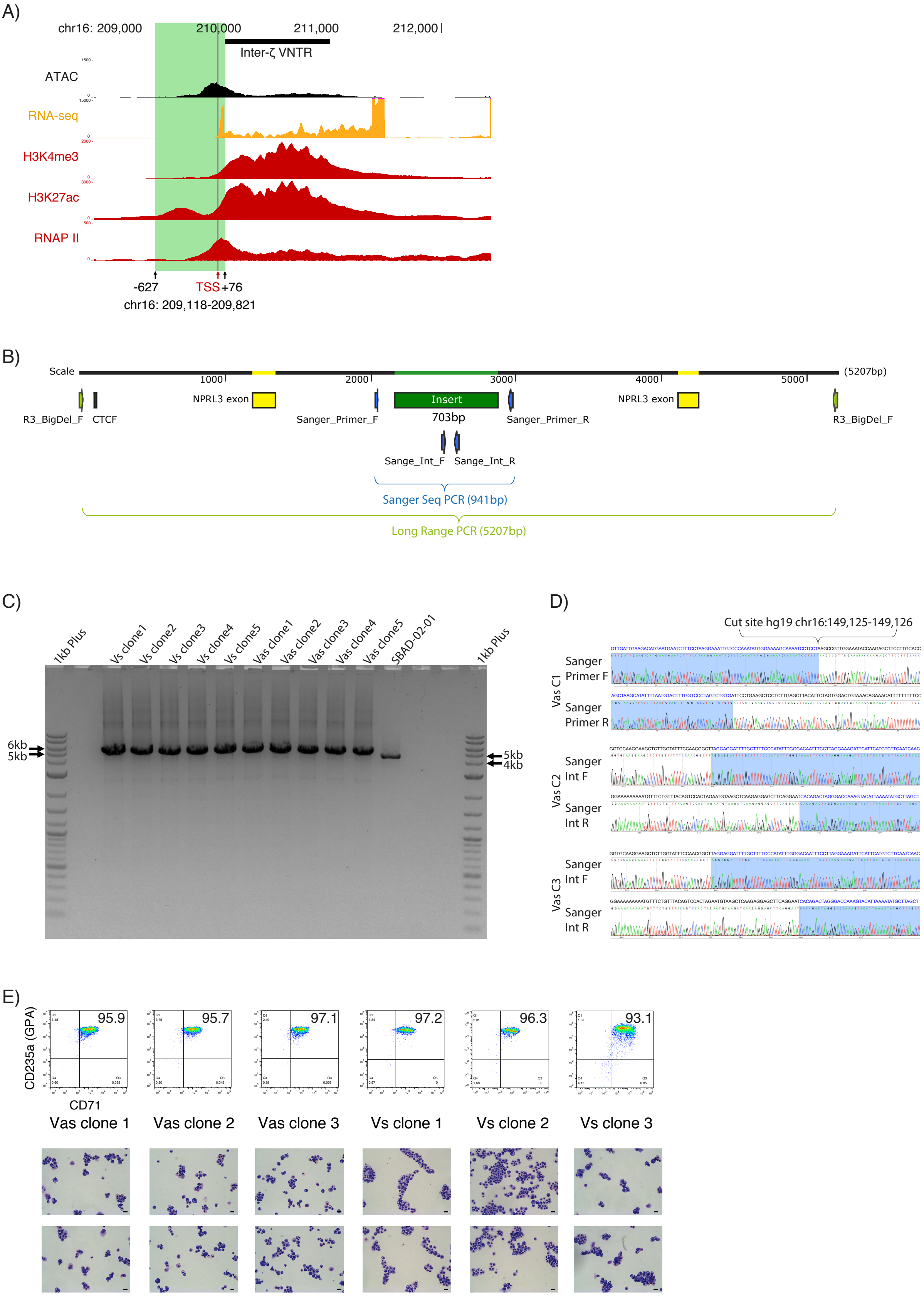

Supplementary figure 3. Novel promoter insertion design and editing quality control.

A) Epigenetic landscape and transcription profile of the site of the novel promoter. The sequence used for transposition is highlighted in green and the distance from the +1 site (labelled as TSS in red; hg19 chr16:209,746) of the *XCPE1* promoter element are shown below. Coordinates (hg19) chr16: 208,000-213,000. **B)** Illustration of a 5207 bp region around the promoter insertion site (hg 19 chr16:146,125-149,126) with promoter insert in dark green and *NPRL3* exons in yellow. The Sanger sequencing PCR fragment (941bp) and associated primers, including internal sequencing primers, are indicated in blue. The long-range PCR fragment (5207bp) and associated primers are indicated in light green. **C)** Long range PCR (primers: R3_BigDel_F + R; ladder: NEB 1kb PLUS) for edited clones (sense insertion - Vs; antisense insertion - Vas) and isogenic control line SBAD-02-01. PCR was replicated twice; representative image is shown. **D)** Sanger sequencing traces for antisense insertion clones, clone name and sequencing primer indicated on the left. **E)** First panel: representative FACS analysis for cell surface iron Transferrin receptor - 1 (CD71) and cell surface glycoprotein Glycophorin A (CD235a or GPA) on day 21 of differentiation. Second panel: modified Wright stain of differentiation culture cells at day 21. Black bar represents 10µm. Each clone was differentiated once. Line names are shown in black.

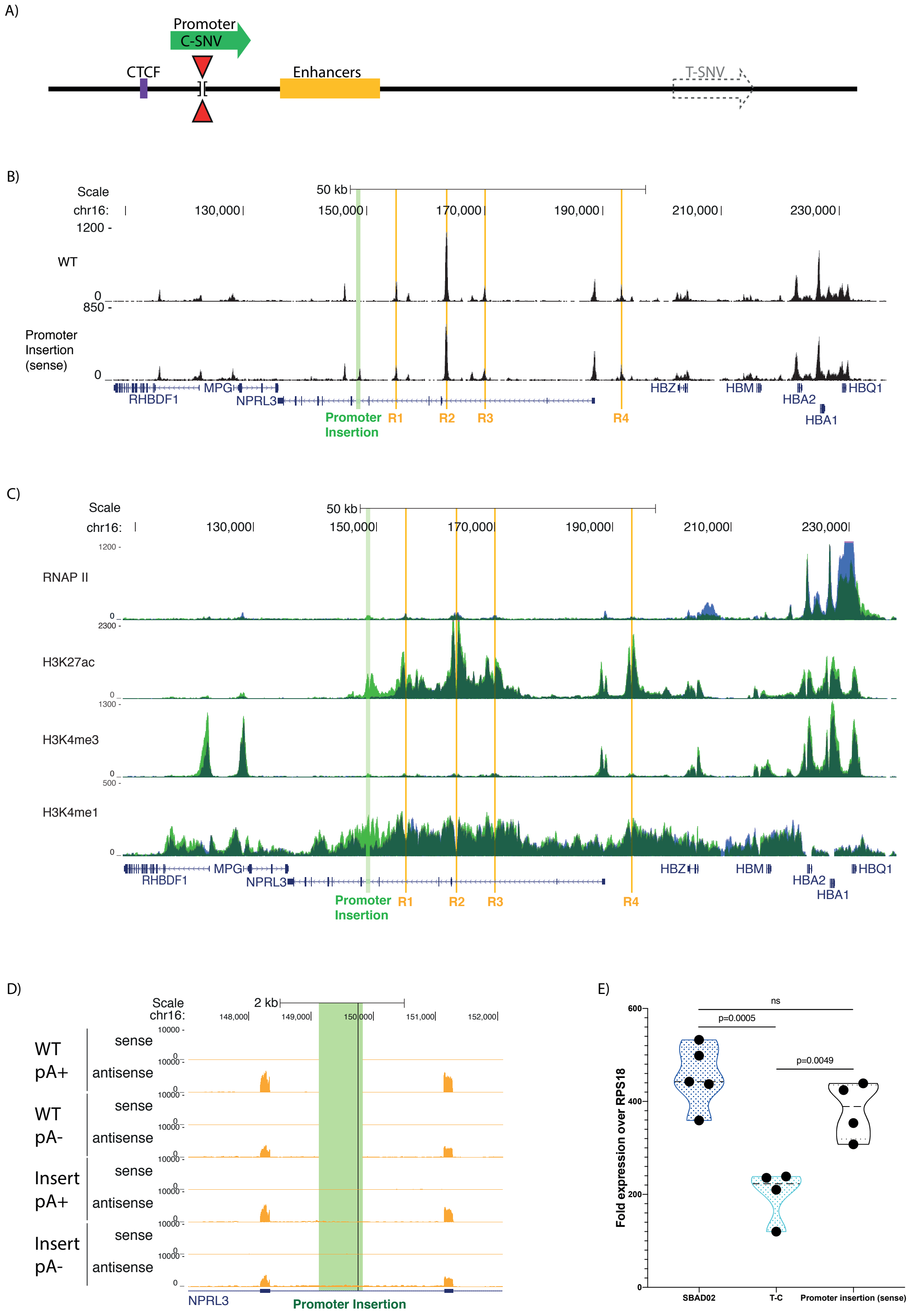

Supplementary figure 4. Placing the novel promoter behind the enhancers in the sense orientation does not produce a promoter element.

A) The active promoter sequence (703bp) was placed behind the α-globin enhancers in the sense orientation. **B)** Chromatin accessibility in the α-globin locus as measured by ATAC-seq (normalised to 100 million reads). The enhancer elements (R1 to R4) are indicated in orange, the inserted promoter is indicated in green (labelled Promoter insertion), gene annotation by Refseq is in blue. Read-densities represent an average of: 3 independent experiments for wild type iPSC line SBAD-02-01 (labelled WT), 3 clones of edited SBAD-02-01 cells where the 703bp of the active promoter sequence is inserted behind R1 in the sense orientation (labelled as Promoter Insertion); all lines differentiated to erythroblasts. Reads were mapped to a custom genome which contains the 703bp promoter insertion in sense. Coordinates (hg19) chr16:108,000-238,000. **C)** ChIP-seq (normalised to 100 million reads), highlighted regions are as in a). Read-densities represent an average of 3 independent experiments for wild type iPSC line SBAD-02-01 (in blue), 3 clones of edited SBAD-02-01 cells where the 703bp of the active promoter sequence is inserted behind R1 in the sense orientation (in green) differentiated to erythroblasts. The tracks are overplayed on top of each other, the darker colour indicates shared signal while green and blue indicate signal unique for the promoter insertion and wild type lines, respectively. Reads were mapped to a custom genome which contains the 703bp promoter insertion in sense. Coordinates (hg19) chr16:108,000-238,000. **D)** Strand-specific RNA-seq of polyadenylated selected (pA+) and non-polyadenylated (pA-) RNA, read density (in RPKM) represents an average of 3 independent experiments, 3 replicates for wild type line SBAD-02-01 or 3 clones of edited SBAD-02-01 cells where the 703bp of the active promoter sequence is inserted behind R1 in the sense orientation differentiated to erythroid cells. The region of the promoter insertion is indicated in green, the TSS is marked by a black line, gene annotation by Refseq is in blue. Coordinates (hg19) chr16:147,000-152,000. **E)** qPCR quantification of HBA1/HBA2 in reference to RPS18 in mRNA obtained from independent differentiation experiments: 5 from wild type iPSC line SBAD-02-01 (WT), 4 clones of edited SBAD-02-01 cells where the T base at position 209,709 (hg19) of chr16 was changed to a C (labelled T - C), 3 clones of edited SBAD-02-01 cells where the 703bp of the active promoter sequence is inserted behind R1 in the sense orientation (labelled as Promoter Insertion (sense)). WT (n=5) in blue, T – C (n=4) in cyan, Promoter Insertion (n=3) in black/white. Violin plots display median (dashed black line) quartile lines (coloured dotted line) and individual data points (black dots). P-values are obtained using unpaired, two-tailed student t-test.

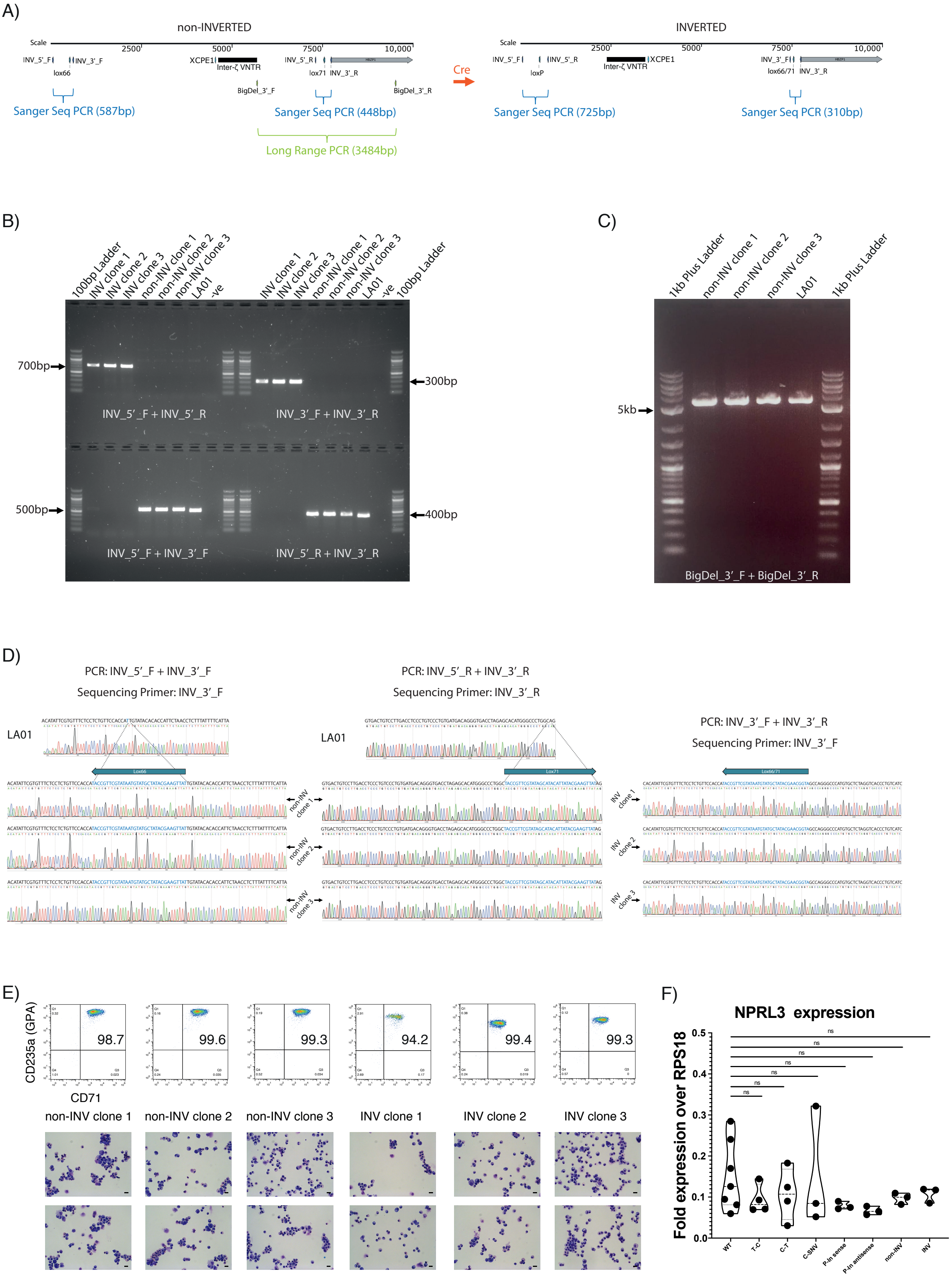

Supplementary figure 5. Novel promoter inversion design and editing quality control.

A) Illustration of a 10kb region around the novel promoter with promoter element XCPE1 in turquoise and heterotypical Lox sites lox66 and lox71 in teal. The Sanger sequencing PCR fragments and associated primers are indicated in blue. The long-

range PCR fragment (3484bp) and associated primers are indicated in light green. Left panel shows the region before inversion and the right panel shows the region after Cre recombinase inverts the segment between the heterotypical Lox sites **B)** PCR genotyping for inversion fragment orientation: inversion produces a 725bp PCR product with primer pair INV_5'_F + INV_5'_R, and a 310bp product with pair INV_3'_F + INV_3'_R; if the fragment isn't inverted (and contains the lox sites) it would produce a 587bp PCR product with primer pair INV_5'_F + INV_3'_F, and a 448bp product with pair INV_5'_R + INV_3'_R. PCRs were performed on lox containing but non-inverted parental clones, on clones obtained after Cre mediated inversion and on isogenic control line LA01. Ladder is 100bp NEB. PCR was replicated twice; representative image is shown. **C)** Long range PCR screen for large deletions was performed at the 3' lox site using primer pair BigDel_3'_F + BigDel_3'_R on lox containing but non-inverted clones and isogenic control parental line LA01. Ladder is 1kb PLUS NEB. PCR was replicated twice; representative image is shown. **D)** Sanger sequencing traces for inverted clones and non-inverted parental clones, clone name and sequencing primer indicated in black. First two panels show traces from the 5' and 3' lox sites of non-inverted parental clone lines and the isogenic (non lox-containing line) control LA01, the last panel shows sequencing traces from the inverted clones at the 3' lox site. After inversion the heterotypical lox66 and lox71 produce a perfect loxP site at the 5' lox site and hybrid lox66/71 site at the 3' lox site with diminished recombination activity towards loxP. **E)** First panel: representative FACS analysis for cell surface iron Transferrin receptor - 1 (CD71) and cell surface glycoprotein Glycophorin A (CD235a or GPA) on day 21 of differentiation. Second panel: modified Wright stain of differentiation culture cells at day 21. Black bar represents 10µm. Each clone was differentiated once. Line names are shown in black. **F)** qPCR quantification of NPRL3 in reference to RPS18 in mRNA obtained from 3 independent wild type iPSC lines (WT), 4 T – C clones (labelled T - C), 4 C – T clones (labelled C - T), 3 iPSC clones obtained from the same patient material (CSNV), 3 clones of Promoter Insertion cells either in sense (P-In sense) or antisense (P-In antisense), 3 clones of non-inverted cells (nonINV), 3 clones of inverted cells (INV) differentiated to erythroblasts. Violin plots display median (dotted black line) quartile lines (coloured dotted line) and individual data points (black dots). P-values are obtained using unpaired, two-tailed student t-test.

Target	Function/Name	Sequence 5' to 3'
HBA2 Promoter Custom TaqMan	Forward primer	GGGCCGGCACTCTTCTG
	Probe	CCCACAGACTCAGAGAGAACCCACCATG
	Reverse primer	GGCCTTGACGTTGGTCTTGT
HS -40 Custom TaqMan	Forward primer	CAGGCTCCAGGCCCATATC
	Probe	TGCCCAAGAGCTCCTTCTGCAACC
	Reverse primer	CCTCCTGCACTGTCCTTTGAC
SNV Custom TaqMan	Forward primer	GGAGGCGGGACACAAGACTA
	Probe	CCTTGCACCAACAGCTTTTCACCTTTCTT
	Reverse primer WT	CAAGCCATTGGAAATACCAAGAG
Negative 14A Custom TaqMan	Forward primer	AACTGCCTTTGCACCCTCAT
	Probe	TGTAGGCAGTCCCCAGTCCCAAAGG
	Reverse primer	GCTTACCTTTTCTGGGCCTACA
Negative 14B Custom TaqMan	Forward primer	CAGCCAATACAATCAGAACAACTGT
	Probe	TTCATGGTATCCTCAAGGCTTGCTGCC
	Reverse primer	GTTTGCGACACTGACTGTGTGA
5' RACE Primer	5' RLM-RACE Primer	AAGAGCTTCCTTGCACCAACAGCT
SNV Sanger Seq PCR	GATAnewF	CATTGTTGAGATGATTTGCTGGAGACACACAGATG
	GATAnewR	GAAATACCAAGAGCTTCCTTGCACCAACAGCT
SNV Long range PCR	LR_GATA_PCR_F	GCTCCTCTTGAGCTTACATTCTAGTGG
	LR_GATA_PCR_R	CAGCACCAAGTCTTTTCTCCCTGTAGCT
SNV Southern Blot Probe	Southern Blot Probe	CTTTCTCTCGGAGCCCTTGGAGAGGGGTATGCAAATATCCGTACTCTAAATATCCTCCATATACTGTGTATTTCCTAAAATCAACAAGGACATTAGGCTGCACAGCCAGAGAACAACCATCAAAATCAGGTTAATATTGATCCAAATCCA
Insertion Sanger Seq PCR	Sanger_Primer_F	ACAAAAGTGGGGAAGGCTCGAAGACT
	Sanger_Primer_R	ACAGAAAGCAAAGCGCTGCCTAGT
Insertion Inner Primers	Sanger_Int_F	CCTCAGGAAGGAAGGGCAGTAGTCA
	Sanger_Int_R	GTCCTCATCTGTGTGTCTCCAGCAAATCATC
Insertion Long Range PCR	R3_BigDel_F	ACGTGCTAAGCAGAGACCTGTTTCACAG
	R3_BigDel_R	TGTGGAGTGAAAGGTTTTCCGTCGTAGT
Lox Site Sanger Seq PCR	INV_5'_F	AGACCTGAAAACCCAGGTGTCCATCATC
	INV_5'_R	GCTCAGAGTGTCCCCTACAAGGCTAC
	INV_3'_F	GTCCACACGCAGCCTCATACATAGTTATGC
	INV_3'_R	CCTATCGCTTCTGTCTCCCCTTCTGC
3' Lox Site Long range PCR	BigDel_3'_F	GGCTTGCAGCTACAGGGAGAAAAGAC
	BigDel_3'_R	CACCTTGAAGTTGACCGGGTCCAC

Target gene ID	Target gene name	TaqMan assay ID
HBA1/2	α-globin	Hs00361191_g1
HBB	β-globin	Hs00747223_g1
HBD	δ-globin	Hs00426283_m1
HBE1	ε-globin	Hs00362216_m1
HBG1/2	γ-globin	Hs00361131_g1
HBZ	ζ-globin	Hs00923579_m1
RPS18	Ribosomal protein S18	Hs01375212_g1

Capture viewpoint	Capture fragment coordinates (hg18)	Probe Sequence
Enhancer R2	chr16: 103,619 -103,765	[Btn]CATGACTCAGTGCTTCTGGAGGCCAACAGGACTGCTGAGTCATCCTGTGGGGGTGGAGGTGGGACAAGGGAAAGGGGTGAATGGTACTGCTGATTACAACCTCTGGTGCTGCCTCCCCCT
		[Btn]AGGACTGCTGAGTCATCCTGTGGGGGTGGAGGTGGGACAAGGGAAAGGGGTGAATGGTACTGCTGATTACAACCTCTGGTGCTGCCTCCCCCTCCTGTTTATCTGAGAGGGAAGGCCATG
SNV fragment	chr16: 149,664-150,998	[Btn]CATGTTGTAGACAGGATGGGTGCTGAGCTGCCACACCCACATTATTAGAAAATAACAGCACAGGCTTGGGGTGGAGGCGGGACACAAGACTAGCCAGAAGGAGAAAGAAAGGTGAAAAGC
		[Btn]TGCAGCTACAGGGAGAAAAGACTTGGTGCTGTGGGCCTGCCTTGGGGCTGGTGGTACAGCCCTTATCTGCTGCCCTCAGGATCTCCCGGCCCCTCTCGTCCAGGCCCCTGCAACCCCATG
HBA1	chr16: 165,923-166,718	[Btn]CATGGTAGCACAGGGCAGCTAAGATGCAAGTCTGAAGGAGGAGTCTGGCGAGCTGCTCCTGCAGTTCCCTGGACCCAGAAAGATGAGCTAGCAGATTCACTTGAGCCAAAGGATTCCAGG
		[Btn]AGAACTGCTCCAAGGGCGTGTCCACCCTGCCTGGAGGACACGCCCTTGGAGGGCATATAAGTGCTACTTGCTGCAGGTCCAAGACACTTCTGATTCTGACAGACTCAGGAAGAAACCATG
HBA2	chr16: 162,119-162,914	[Btn]CATGGTAGCACAGGGCAGCTAAGATGCAAGTCTGAAGGAGGAGTCTGGCGAGCTGCTCCTGCAGTTCCCTGGACCCAGAAGGATGAGCTAGCAGATTCACTTGAGCCAAAGGATTCCAGG
		[Btn]AGAACTGCTCCAAGGGCGTGTCCACCCTGCCTGGAGGACACGCCCTTGGAGGGCATATAAGTGCTACTTGCTGCAGGTCCAAGACACTTCTGATTCTGACAGACTCAGGAAGAAACCATG

Editing primers and ssODN	Function	Oligo Sequence
sgRNA recognising T allele of C-SNV	T-C sgRNA sense cloning oligo for PX459 V2.0	caccGTAT**T**AGAAAATAACAGCAC
	T-C sgRNA antisense cloning oligo for PX459 V2.0	aaacGTGCTGTTATTTTCT**A**ATAC
sgRNA recognising C allele of C-SNV	C-T sgRNA sense cloning oligo for PX459 V2.0	caccGTAT**C**AGAAAATAACAGCAC
	C-T sgRNA antisense cloning oligo for PX459 V2.0	aaacGTGCTGTTATTTTCT**G**ATAC
ssODN repair template for T to C edit	T-C ssODN asymetric repair template	GAAATACCAAGAGCTTCCTTGCACCAACAGCTTTTCACCTTTCTTTCTCCTTCTGGCTAGTCTTGTGTCCCGCCTCCACCCCAAGCCTGTGCTGTTATTTTC**TGATAA**TGTGGGTGTGGCAGCTCAG
ssODN repair template for C to T edit	C-T ssODN asymetric repair template	GAAATACCAAGAGCTTCCTTGCACCAACAGCTTTTCACCTTTCTTTCTCCTTCTGGCTAGTCTTGTGTCCCGCCTCCACCCCAAGCCTGTGCTGTTATTTTC**TAATAA**TGTGGGTGTGGCAGCTCAG
sgRNA recognising promoter insertion site	Promoter insertion sgRNA sense cloning oligo for PX459 V2.0	caccGGTGGTCCCTAGTCTGTGAGG
	Promoter insertion sgRNA antisense cloning oligo for PX459 V2.0	aaacCCTCACAGACTAGGGACCACC
ssODN sequence for promoter isertion in antisense (Vas)	Vas long ssODN repair template with 60bp homology arms	GACATGAATGAATCTTTCCTAAGGAAATTGTCCCAAATATGGGAAAAGCAAAATCCTCCT**AAGCCGTTGGAAATACCAAGAGCTTCCTTGCACCAACAGCTTTTCACCTTTCTTTCTGCTTCTGGCTAGTCTTGTGTCCCGCCTCCACCCCAAGCCTGTGCTGTTATTTTCTGATAATGTGGGTGTGGCAGCTCAGCACCCATCCTGTCTACAACATGACATTAGCCTCCATCTGTGTCCTGGTGCTCCCATTGATGGGTGTGTCCATACAGACCTCTCTTCCGGACTCAGGCCCCAGGGCCTGATCCAGGGATGTATTTGATGTCCTCATCTGTGTGTCTCCAGCAAATCATCTCAACAATGGAACCGTGAAGTTCCCCACAAAGCCTACTCCACTGTCCTTCCCTGGCATCCCTGACTACTGCCCTTCCTTCCTGAGGCTCAGGCCGGAGAGCACAGCACCCTCGACACCCCCCACTCACTCCTGAAACTTACATATGGAGGAAAAGACAGACAAGGCCTCCTGTCCTCCCTCCCTCTATCCCTAGCTCCTGGCCACTCCCCTGAAGCCCCTTCCCTAGTATTGCCACTCCCTCAAATGTCACCACGTTAATAGACCTACAGTGGCCAACCCATTTAAAATCTGAATCCTCCCCTCCTCTGTCTAAGCCCCTACTTGCCCTGCTTTTCTTTATCCACAGGAAAAAAAAATGTTTCTGTTTACAGTCCACTAGAATGTAAGCTCAAGAGGAGCTTCAGGAA**TCACAGACTAGGGACCAAAGTACATTAAAATATGCTTAGCTAGGATTGAAAGCAAAGAGCA
ssODN sequence for promoter isertion in sense (Vs)	Vs long ssODN repair template with 60bp homology arms	GACATGAATGAATCTTTCCTAAGGAAATTGTCCCAAATATGGGAAAAGCAAAATCCTCCTA**TTCCTGAAGCTCCTCTTGAGCTTACATTCTAGTGGACTGTAAACAGAAACATTTTTTTTTCCTGTGGATAAAGAAAAGCAGGGCAAGTAGGGGCTTAGACAGAGGAGGGGAGGATTCAGATTTTAAATGGGTTGGCCACTGTAGGTCTATTAACGTGGTGACATTTGAGGGAGTGGCAATACTAGGGAAGGGGCTTCAGGGGAGTGGCCAGGAGCTAGGGATAGAGGGAGGGAGGACAGGAGGCCTTGTCTGTCTTTTCCTCCATATGTAAGTTTCAGGAGTGAGTGGGGGGTGTCGAGGGTGCTGTGCTCTCCGGCCTGAGCCTCAGGAAGGAAGGGCAGTAGTCAGGGATGCCAGGGAAGGACAGTGGAGTAGGCTTTGTGGGGAACTTCACGGTTCCATTGTTGAGATGATTTGCTGGAGACACACAGATGAGGACATCAAATACATCCCTGGATCAGGCCCTGGGGCCTGAGTCCGGAAGAGAGGTCTGTATGGACACACCCATCAATGGGAGCACCAGGACACAGATGGAGGCTAATGTCATGTTGTAGACAGGATGGGTGCTGAGCTGCCACACCCACATTATCAGAAAATAACAGCACAGGCTTGGGGTGGAGGCGGGACACAAGACTAGCCAGAAGCAGAAAGAAAGGTGAAAAGCTGTTGGTGCAAGGAAGCTCTTGGTATTTCCAACGGCTT**CACAGACTAGGGACCAAAGTACATTAAAATATGCTTAGCTAGGATTGAAAGCAAAGAGCA
sgRNA recognising 5' Lox insertion site	5' Lox66 insertion sgRNA sense cloning oligo for PX459 V2.0	caccGGAATGGTGTGTATACAATGG
	5' Lox66 insertion sgRNA antisense cloning oligo for PX459 V2.0	aaacCCATTGTATACACACCATTCC
sgRNA recognising 3' Lox insertion site	3' Lox71 insertion sgRNA sense cloning oligo for PX459 V2.0	caccGGAATGGATCGTCCCTCTGCC
	3' Lox71 insertion sgRNA antisense cloning oligo for PX459 V2.0	aaacGGCAGAGGGACGATCCATTCC
ssODN repair template for 5' Lox insertion	5' Lox66 ssODN asymetric repair template	CACACATGTCCACACGCAGCCTCATACATAGTTATGCATAAGCTCACATATATGTGCACACACATATTCGTGTTTCTCCTCTGTTCCACCA**TACCGTTCGTATAATGTATGCTATACGAAGTTAT**TGTATACACACCATTCTAACCTCTTTATTTTCATT
ssODN repair template for 3' Lox insertion	3' Lox71 ssODN asymetric repair template	TCAGCCCAGCCCGGACTGGCCTCAGGAGTGACTGTCCTTGACCTCCCTGTCCCTGTGATGACAGGGTGACCTAGAGCACATGGGCCCTGGC**TACCGTTCGTATAGCATACATTATACGAAGTTAT**AGAGGGACGATCCATTCCTCACTTCACTGAAACTTC

Supplementary table 1. Oligo and probe sequences and catalog numbers
