# Supplementary material for: A gain-of-function single nucleotide variant creates a new promoter which acts as an orientation-dependent enhancer-blocker
Source: Nat Commun. 2021 Jun 21;12:3806. doi: 10.1038/s41467-021-23980-6 (PMC8217497; doi:10.1038/s41467-021-23980-6)
Supplement: Supplementary file 2 — Reporting Summary [file 41467_2021_23980_MOESM2_ESM.pdf]

nature research

Corresponding author(s): Douglas Higgs

Last updated by author(s): Feb 22, 2021

Reporting Summary

Nature Research wishes to improve the reproducibility of the work that we publish. This form provides structure for consistency and transparency in reporting. For further information on Nature Research policies, see our Editorial Policies and the Editorial Policy Checklist.

Statistics

For all statistical analyses, confirm that the following items are present in the figure legend, table legend, main text, or Methods section.

n/a	Confirmed	
[ ]	[x]	The exact sample size (*n*) for each experimental group/condition, given as a discrete number and unit of measurement
[ ]	[x]	A statement on whether measurements were taken from distinct samples or whether the same sample was measured repeatedly
[ ]	[x]	The statistical test(s) used AND whether they are one- or two-sided<br>*Only common tests should be described solely by name; describe more complex techniques in the Methods section.*
[ ]	[x]	A description of all covariates tested
[ ]	[x]	A description of any assumptions or corrections, such as tests of normality and adjustment for multiple comparisons
[ ]	[x]	A full description of the statistical parameters including central tendency (e.g. means) or other basic estimates (e.g. regression coefficient) AND variation (e.g. standard deviation) or associated estimates of uncertainty (e.g. confidence intervals)
[ ]	[x]	For null hypothesis testing, the test statistic (e.g. *F*, *t*, *r*) with confidence intervals, effect sizes, degrees of freedom and *P* value noted<br>*Give P values as exact values whenever suitable.*
[x]	[ ]	For Bayesian analysis, information on the choice of priors and Markov chain Monte Carlo settings
[x]	[ ]	For hierarchical and complex designs, identification of the appropriate level for tests and full reporting of outcomes
[x]	[ ]	Estimates of effect sizes (e.g. Cohen's *d*, Pearson's *r*), indicating how they were calculated

Our web collection on statistics for biologists contains articles on many of the points above.

Software and code

Policy information about availability of computer code

Data collection	none
Data analysis	Several previously published software packages were used for the analysis of genome-wide data. These include FASTQC 0.11.9, Bowtie 2.3.2, Samtools 0.1.19, Bedtools 2.25.0, Deeptools 2.2.2. For ChIP-seq and ATAC-seq, data analysis was performed using an in-house pipeline: https://github.com/Hughes-Genome-Group/NGseqBasic/releases. RNA-seq analysis: read QC analysis with the fastQC package (http://www.bioinformatics.babraham.ac.uk/projects/fastqc) and reads were aligned using STAR 2.7.4. PCR duplicates were discarded using Samtools, normalization to RPKM was performed using deeptools. Capture-C analysis was performed using an in-house pipeline, which can be found at https://github.com/Hughes-Genome-Group/CCseqBasicF/releases. The resulting tracks were run through the CaptureSee tool (https://capturesee.molbiol.ox.ac.uk/) as described in Telenius et al. 2020. Copy number variation analysis for data obtained from the Infinium HD assay (Human CytoSNP 12 Beadchip v2.1, Illumina) was performed using the KaryoStudio 1.4.3 software package. FACS data was analyzed using FlowJo 10.6.2 software.

For manuscripts utilizing custom algorithms or software that are central to the research but not yet described in published literature, software must be made available to editors and reviewers. We strongly encourage code deposition in a community repository (e.g. GitHub). See the Nature Research guidelines for submitting code & software for further information.

Data

Policy information about availability of data

All manuscripts must include a data availability statement. This statement should provide the following information, where applicable:

- Accession codes, unique identifiers, or web links for publicly available datasets
- A list of figures that have associated raw data
- A description of any restrictions on data availability

ATAC-seq, ChIP-Seq, RNA-seq and NG Capture-C raw data and bigwig are available under Gene Expression Omnibus (GEO) accession GSE159875. Analyses and coordinates referenced here are for either the hg19, hg18 human reference genomes, or custom genomes hg19_INV (inverted C-SNV promoter), hg19_Vas (promoter insertion in anti-sense behind enhancers) or hg19_Vs (promoter insertion in sense behind enhancers) based on reference human genome hg19, as indicated in figure legends. Sequences for chromosome 16 from the custom genomes are available in FASTA format as supplementary files in GEO Subseries GSE159871. Figures that have associated raw data files: fig.1 a), b), c); fig.2 c); fig.3 a), b), c), d); fig.4 b), c), d), f); 5 b), c), d), f); supp. fig 4 b), c), d);

Field-specific reporting

Please select the one below that is the best fit for your research. If you are not sure, read the appropriate sections before making your selection.

☒ Life sciences ☐ Behavioural & social sciences ☐ Ecological, evolutionary & environmental sciences

For a reference copy of the document with all sections, see nature.com/documents/nr-reporting-summary-flat.pdf

Life sciences study design

All studies must disclose on these points even when the disclosure is negative.

Sample size	Sample sizes were selected to allow robust statistical analysis. No statistical method was used to predetermine sample size, experiments were performed in at least 3 biological replicates for RT-qPCR, ATAC-seq and Capture C and at least 2 replicates for ChIP-seq as is common in the field and the observed biological effects of interest were very robust between replicates. Number of replicates is mentioned where relevant in figure legends.
Data exclusions	One differentiation replicate (of two) of line LA06 was removed from the RT-qPCR and subsequent analysis due to overall low levels of expression (high Ct values).
Replication	Gene expression experiments by RT-qPCR have 3-6 independent replicates with similar results. Capture-C experiments were performed in independent biological triplicate with similar results. ChIP and RNA-seq experiments were performed at least in duplicate with similar results. ATAC-seq experiments were performed at least in triplicate with similar results.
Randomization	Experiments were not randomized. Experimental groups of cells were selected by genotype.
Blinding	Investigators were not blinded during experiments or analysis. Blinding was not relevant to this study as the data presented are objectively obtained by quantification of cellular phenotypes through measurements of mRNA levels, protein levels and chromatin interactions and all samples were analyzed using the same pipelines and/or scripts. Experimental groups of cells were selected by genotype.

Reporting for specific materials, systems and methods

We require information from authors about some types of materials, experimental systems and methods used in many studies. Here, indicate whether each material, system or method listed is relevant to your study. If you are not sure if a list item applies to your research, read the appropriate section before selecting a response.

Materials & experimental systems

n/a	Involved in the study	
☐	☒	Antibodies
☐	☒	Eukaryotic cell lines
☒	☐	Palaeontology and archaeology
☒	☐	Animals and other organisms
☒	☐	Human research participants
☒	☐	Clinical data
☒	☐	Dual use research of concern

Methods

n/a	Involved in the study	
☐	☒	ChIP-seq
☐	☒	Flow cytometry
☒	☐	MRI-based neuroimaging

Antibodies

Antibodies used	RNA Polymerase II N-20 antibody SC-899 (Santa Cruz Biotechnology) 6µg per 1ml of chromatin from 1x10^7 cells;<br>Monoclonal anti-acetyl-Histone H3 (Lys27) antibody 17-683 (Merck Millipore) 2µg per 1ml of chromatin from 1x10^7 cells;<br>Polyclonal anti-trimethyl-Histone H3 (Lys4) antibody 07-473 (Merck Millipore) 1µg per 1ml of chromatin from 1x10^7 cells;<br>Polyclonal antimonomethyl-Histone H3 (Lys4) antibody ab195391 (Abcam) 4µg per 1ml of chromatin from 1x10^7 cells;<br>Polyclonal anti-GATA1 antibody ab11852 (Abcam) 8µg per 1ml of chromatin from 1x10^7 cells;<br>Polyclonal anti-CTCF antibody 07-729 (Merck Millipore) 10µl serum per 1ml of chromatin from 1x10^7 cells<br>Polyclonal anti-KLF1 antibody was kindly provided by the Perkins Laboratory, Translational Research Institute, Brisbane, Australia<br>FITC conjugated anti-CD71, 555536 (BD Biosciences) 1:200 dilution<br>PE conjugated anti-CD235a, 340947 (BD Biosciences) 1:200 dilution
Validation	SC-899 (Santa Cruz Biotechnology) - Anti-Pol II Antibody (8WG16) with reactivity against the highly conserved heptapeptide repeat of the largest subunit of eukaryotic Pol II of mouse, rat, human, Xenopus, C. elegans, yeast, wheat germ and bovine origin. Validated by manufacturer using WB on HeLa nuclear extract. Used for ChIP experiments in human cells in 37 publications as of 25.03.2021 (for ref. see https://www.citeab.com/antibodies/825104-sc-899-pol-ii-n-20)<br><br>17-683 (Merck Millipore) - Anti-acetyl-Histone H3 (Lys27) Antibody (mouse monoclonal IgG1, Clone CMA309)made against a synthetic peptide (acetylated at Lys27) corresponding to amino acids 19-37 of histone H3. Reactivity against a wide range of vertebrate species including human and mouse. Validated by manufacturer using ChIP on sonicated chromatin prepared from HeLa cells: 37 fold enrichment vs normal mouse IgG at target site. Used for ChIP experiments in 8 publications as of 25.03.2021 (for ref. see https://www.citeab.com/antibodies/222510-17-683-chipab-acetyl-histone-h3-lys27-chip-valid?des=1b1830e4de86bbf7)<br><br>07-473 (Merck Millipore) - Anti-trimethylated-Histone H3 (Lys4) Antibody made against a synthetic peptide corresponding to residues surrounding and including trimethlyated Lys 4 of Histone H3. Reactivity against a wide range of vertebrate species including human. Validated by manufacturer using WB on HeLa nuclear extract. Validated by an independent laboratory for ChIP-seq as quoted by manufacturers website. Used for ChIP experiments in human cells in 41 publications as of 25.03.2021 (for ref. see https://www.citeab.com/antibodies/221386-07-473-anti-trimethyl-histone-h3-lys4-antibody?des=f2f6563974e6d703)<br><br>ab195391 (Abcam) - Anti-monomethyl-Histone H3 (K4) Antibody made against synthetic peptide within Human Histone H3 (mono methyl K4) conjugated to keyhole limpet haemocyanin. Reactivity against a wide range of vertebrate species including human. Validated by manufacturer using ChIP on K562 nuclear extract: over 20 fold enrichment vs normal mouse IgG at target site. For reference see: https://www.abcam.com/histone-h3-mono-methyl-k4-antibody-chip-grade-ab195391.html<br><br>ab11852 (Abcam) - Anti-GATA1 Antibody made against a synthetic peptide corresponding to amino acids 394-413 of Human GATA1. Reactivity against human. Validated by manufacturer using WB on K562 nuclear extract. Used for ChIP experiments in human cells in 4 publications as of 25.03.2021 (for ref. see https://www.citeab.com/antibodies/732427-ab11852-anti-gata1-antibody-chip-grade?des=32daec65a62b26b8)<br><br>07-729 (Merck Millipore) - Anti-GATA1 Antibody made against a synthetic peptide corresponding to amino acids 659-675 of human CCCTC-binding factor (CTCF).Reactivity against a wide range of vertebrate species including human and mouse. Validated by manufacturer using WB on K562 nuclear extract.Used for ChIP experiments in human cells in 21 publications as of 25.03.2021 (for ref. see https://www.citeab.com/antibodies/221655-07-729-anti-ctcf-antibody?des=22d6cb54f54c5584)<br><br>anti-KLF1 antibody validated at provider laboratory (Hodge et al., 2006)

Eukaryotic cell lines

Policy information about cell lines

Cell line source(s)	SB-AD2-01 wild type iPSC line obtained from Cader laboratory (WIMM, Oxford) via StemBANCC (Oxford)<br>SB-AD3-01 wild type iPSC line obtained from Cader laboratory (WIMM, Oxford) via StemBANCC (Oxford)<br>AH017-13 wild type iPSC line obtained from Cader laboratory (WIMM, Oxford) via StemBANCC (Oxford)<br>Lines LA01, LA06, LA13 were generated and validated in house.
Authentication	All lines were tested for expression of pluripotentcy factors and were assessed for the presence of large CNVs using SNP array.
Mycoplasma contamination	All lines were routinely tested (every 6 months) for mycoplasma contamination and were tested each time before sample collection and were always negative.
Commonly misidentified lines<br>(See ICLAC register)	none

ChIP-seq

Data deposition

☒ Confirm that both raw and final processed data have been deposited in a public database such as GEO.

☒ Confirm that you have deposited or provided access to graph files (e.g. BED files) for the called peaks.

Data access links<br>*May remain private before publication.*	https://www.ncbi.nlm.nih.gov/geo/query/acc.cgi?acc=GSE159875
Files in database submission	ChIP_CTCF_Ery_iPSC_C-SNV_LA01_rep1.bw<br>ChIP_CTCF_Ery_iPSC_C-SNV_LA06_rep1.bw<br>ChIP_CTCF_Ery_iPSC_C-SNV_LA13_rep1.bw<br>ChIP_CTCF_Ery_iPSC_WT_AH017_rep1.bw<br>ChIP_CTCF_Ery_iPSC_WT_AH017_rep2.bw<br>ChIP_CTCF_Ery_iPSC_WT_AH017_rep3.bw<br>ChIP_H3K27ac_Ery_iPSC_C-SNV_LA01_rep1.bw<br>ChIP_H3K27ac_Ery_iPSC_C-SNV_LA06_rep1.bw<br>ChIP_H3K27ac_Ery_iPSC_C-SNV_LA13_rep1.bw<br>ChIP_H3K27ac_Ery_iPSC_Vas_Clone1_rep1.bw<br>ChIP_H3K27ac_Ery_iPSC_Vas_Clone2_rep1.bw<br>ChIP_H3K27ac_Ery_iPSC_Vas_Clone3_rep1.bw<br>ChIP_H3K27ac_Ery_iPSC_Vs_Clone1_rep1.bw<br>ChIP_H3K27ac_Ery_iPSC_Vs_Clone2_rep1.bw<br>ChIP_H3K27ac_Ery_iPSC_Vs_Clone3_rep1.bw<br>ChIP_H3K27ac_Ery_iPSC_WT_AH017_rep1.bw<br>ChIP_H3K27ac_Ery_iPSC_WT_AH017_rep2.bw<br>ChIP_H3K27ac_Ery_iPSC_WT_AH017_rep3.bw<br>ChIP_H3K27ac_Ery_iPSC_WT_SBAD02_rep1.bw<br>ChIP_H3K27ac_Ery_iPSC_WT_SBAD02_rep2.bw<br>ChIP_H3K27ac_Ery_iPSC_WT_SBAD02_rep3.bw<br>ChIP_H3K4me1_Ery_iPSC_C-SNV_LA01_rep1.bw<br>ChIP_H3K4me1_Ery_iPSC_C-SNV_LA06_rep1.bw<br>ChIP_H3K4me1_Ery_iPSC_C-SNV_LA13_rep1.bw<br>ChIP_H3K4me1_Ery_iPSC_Vas_Clone1_rep1.bw<br>ChIP_H3K4me1_Ery_iPSC_Vas_Clone2_rep1.bw<br>ChIP_H3K4me1_Ery_iPSC_Vas_Clone3_rep1.bw<br>ChIP_H3K4me1_Ery_iPSC_Vs_Clone1_rep1.bw<br>ChIP_H3K4me1_Ery_iPSC_Vs_Clone2_rep1.bw<br>ChIP_H3K4me1_Ery_iPSC_Vs_Clone3_rep1.bw<br>ChIP_H3K4me1_Ery_iPSC_WT_AH017_rep1.bw<br>ChIP_H3K4me1_Ery_iPSC_WT_AH017_rep2.bw<br>ChIP_H3K4me1_Ery_iPSC_WT_AH017_rep3.bw<br>ChIP_H3K4me1_Ery_iPSC_WT_SBAD02_rep1.bw<br>ChIP_H3K4me1_Ery_iPSC_WT_SBAD02_rep2.bw<br>ChIP_H3K4me1_Ery_iPSC_WT_SBAD02_rep3.bw<br>ChIP_H3K4me3_Ery_iPSC_C-SNV_LA01_rep1.bw<br>ChIP_H3K4me3_Ery_iPSC_C-SNV_LA06_rep1.bw<br>ChIP_H3K4me3_Ery_iPSC_C-SNV_LA13_rep1.bw<br>ChIP_H3K4me3_Ery_iPSC_INV_Clone1_rep1.bw<br>ChIP_H3K4me3_Ery_iPSC_INV_Clone2_rep1.bw<br>ChIP_H3K4me3_Ery_iPSC_Vas_Clone1_rep1.bw<br>ChIP_H3K4me3_Ery_iPSC_Vas_Clone2_rep1.bw<br>ChIP_H3K4me3_Ery_iPSC_Vas_Clone3_rep1.bw<br>ChIP_H3K4me3_Ery_iPSC_Vs_Clone1_rep1.bw<br>ChIP_H3K4me3_Ery_iPSC_Vs_Clone2_rep1.bw<br>ChIP_H3K4me3_Ery_iPSC_Vs_Clone3_rep1.bw<br>ChIP_H3K4me3_Ery_iPSC_WT_AH017_rep1.bw<br>ChIP_H3K4me3_Ery_iPSC_WT_AH017_rep2.bw<br>ChIP_H3K4me3_Ery_iPSC_WT_AH017_rep3.bw<br>ChIP_H3K4me3_Ery_iPSC_WT_SBAD02_rep1.bw<br>ChIP_H3K4me3_Ery_iPSC_WT_SBAD02_rep2.bw<br>ChIP_H3K4me3_Ery_iPSC_WT_SBAD02_rep3.bw<br>ChIP_RNAPII_Ery_iPSC_C-SNV_LA01_rep1.bw<br>ChIP_RNAPII_Ery_iPSC_C-SNV_LA06_rep1.bw<br>ChIP_RNAPII_Ery_iPSC_C-SNV_LA13_rep1.bw<br>ChIP_RNAPII_Ery_iPSC_INV_Clone1_rep1.bw

ChIP_RNAPII_Ery_iPSC_INV_Clone2_rep1.bw
ChIP_RNAPII_Ery_iPSC_Vs_Clone1_rep1.bw
ChIP_RNAPII_Ery_iPSC_Vs_Clone2_rep1.bw
ChIP_RNAPII_Ery_iPSC_Vs_Clone3_rep1.bw
ChIP_RNAPII_Ery_iPSC_WT_AH017_rep1.bw
ChIP_RNAPII_Ery_iPSC_WT_AH017_rep2.bw
ChIP_RNAPII_Ery_iPSC_WT_AH017_rep3.bw
ChIP_RNAPII_Ery_iPSC_WT_SBAD02_rep1.bw
ChIP_RNAPII_Ery_iPSC_WT_SBAD02_rep2.bw
ChIP_RNAPII_Ery_iPSC_WT_SBAD02_rep3.bw
ChIP_CTCF_Ery_iPSC_C-SNV_LA01_rep1_R1.fastq.gz
ChIP_CTCF_Ery_iPSC_C-SNV_LA01_rep1_R2.fastq.gz
ChIP_CTCF_Ery_iPSC_C-SNV_LA06_rep1_R1.fastq.gz
ChIP_CTCF_Ery_iPSC_C-SNV_LA06_rep1_R2.fastq.gz
ChIP_CTCF_Ery_iPSC_C-SNV_LA13_rep1_R1.fastq.gz
ChIP_CTCF_Ery_iPSC_C-SNV_LA13_rep1_R2.fastq.gz
ChIP_CTCF_Ery_iPSC_WT_AH017_rep1_R1.fastq.gz
ChIP_CTCF_Ery_iPSC_WT_AH017_rep1_R2.fastq.gz
ChIP_CTCF_Ery_iPSC_WT_AH017_rep2_R1.fastq.gz
ChIP_CTCF_Ery_iPSC_WT_AH017_rep2_R2.fastq.gz
ChIP_CTCF_Ery_iPSC_WT_AH017_rep3_R1.fastq.gz
ChIP_CTCF_Ery_iPSC_WT_AH017_rep3_R2.fastq.gz
ChIP_H3K27ac_Ery_iPSC_C-SNV_LA01_rep1_R1.fastq.gz
ChIP_H3K27ac_Ery_iPSC_C-SNV_LA01_rep1_R2.fastq.gz
ChIP_H3K27ac_Ery_iPSC_C-SNV_LA06_rep1_R1.fastq.gz
ChIP_H3K27ac_Ery_iPSC_C-SNV_LA06_rep1_R2.fastq.gz
ChIP_H3K27ac_Ery_iPSC_C-SNV_LA13_rep1_R1.fastq.gz
ChIP_H3K27ac_Ery_iPSC_C-SNV_LA13_rep1_R2.fastq.gz
ChIP_H3K27ac_Ery_iPSC_Vas_Clone1_rep1_R1.fastq.gz
ChIP_H3K27ac_Ery_iPSC_Vas_Clone1_rep1_R2.fastq.gz
ChIP_H3K27ac_Ery_iPSC_Vas_Clone2_rep1_R1.fastq.gz
ChIP_H3K27ac_Ery_iPSC_Vas_Clone2_rep1_R2.fastq.gz
ChIP_H3K27ac_Ery_iPSC_Vas_Clone3_rep1_R1.fastq.gz
ChIP_H3K27ac_Ery_iPSC_Vas_Clone3_rep1_R2.fastq.gz
ChIP_H3K27ac_Ery_iPSC_Vs_Clone1_rep1_R1.fastq.gz
ChIP_H3K27ac_Ery_iPSC_Vs_Clone1_rep1_R2.fastq.gz
ChIP_H3K27ac_Ery_iPSC_Vs_Clone2_rep1_R1.fastq.gz
ChIP_H3K27ac_Ery_iPSC_Vs_Clone2_rep1_R2.fastq.gz
ChIP_H3K27ac_Ery_iPSC_Vs_Clone3_rep1_R1.fastq.gz
ChIP_H3K27ac_Ery_iPSC_Vs_Clone3_rep1_R2.fastq.gz
ChIP_H3K27ac_Ery_iPSC_WT_AH017_rep1_R1.fastq.gz
ChIP_H3K27ac_Ery_iPSC_WT_AH017_rep1_R2.fastq.gz
ChIP_H3K27ac_Ery_iPSC_WT_AH017_rep2_R1.fastq.gz
ChIP_H3K27ac_Ery_iPSC_WT_AH017_rep2_R2.fastq.gz
ChIP_H3K27ac_Ery_iPSC_WT_AH017_rep3_R1.fastq.gz
ChIP_H3K27ac_Ery_iPSC_WT_AH017_rep3_R2.fastq.gz
ChIP_H3K27ac_Ery_iPSC_WT_SBAD02_rep1_R1.fastq.gz
ChIP_H3K27ac_Ery_iPSC_WT_SBAD02_rep1_R2.fastq.gz
ChIP_H3K27ac_Ery_iPSC_WT_SBAD02_rep2_R1.fastq.gz
ChIP_H3K27ac_Ery_iPSC_WT_SBAD02_rep2_R2.fastq.gz
ChIP_H3K27ac_Ery_iPSC_WT_SBAD02_rep3_R1.fastq.gz
ChIP_H3K27ac_Ery_iPSC_WT_SBAD02_rep3_R2.fastq.gz
ChIP_H3K4me1_Ery_iPSC_C-SNV_LA01_rep1_R1.fastq.gz
ChIP_H3K4me1_Ery_iPSC_C-SNV_LA01_rep1_R2.fastq.gz
ChIP_H3K4me1_Ery_iPSC_C-SNV_LA06_rep1_R1.fastq.gz
ChIP_H3K4me1_Ery_iPSC_C-SNV_LA06_rep1_R2.fastq.gz
ChIP_H3K4me1_Ery_iPSC_C-SNV_LA13_rep1_R1.fastq.gz
ChIP_H3K4me1_Ery_iPSC_C-SNV_LA13_rep1_R2.fastq.gz
ChIP_H3K4me1_Ery_iPSC_Vas_Clone1_rep1_R1.fastq.gz
ChIP_H3K4me1_Ery_iPSC_Vas_Clone1_rep1_R2.fastq.gz
ChIP_H3K4me1_Ery_iPSC_Vas_Clone2_rep1_R1.fastq.gz
ChIP_H3K4me1_Ery_iPSC_Vas_Clone2_rep1_R2.fastq.gz
ChIP_H3K4me1_Ery_iPSC_Vas_Clone3_rep1_R1.fastq.gz
ChIP_H3K4me1_Ery_iPSC_Vas_Clone3_rep1_R2.fastq.gz
ChIP_H3K4me1_Ery_iPSC_Vs_Clone1_rep1_R1.fastq.gz
ChIP_H3K4me1_Ery_iPSC_Vs_Clone1_rep1_R2.fastq.gz
ChIP_H3K4me1_Ery_iPSC_Vs_Clone2_rep1_R1.fastq.gz
ChIP_H3K4me1_Ery_iPSC_Vs_Clone2_rep1_R2.fastq.gz

ChIP_H3K4me1_Ery_iPSC_Vs_Clone3_rep1_R1.fastq.gz
ChIP_H3K4me1_Ery_iPSC_Vs_Clone3_rep1_R2.fastq.gz
ChIP_H3K4me1_Ery_iPSC_WT_AH017_rep1_R1.fastq.gz
ChIP_H3K4me1_Ery_iPSC_WT_AH017_rep1_R2.fastq.gz
ChIP_H3K4me1_Ery_iPSC_WT_AH017_rep2_R1.fastq.gz
ChIP_H3K4me1_Ery_iPSC_WT_AH017_rep2_R2.fastq.gz
ChIP_H3K4me1_Ery_iPSC_WT_AH017_rep3_R1.fastq.gz
ChIP_H3K4me1_Ery_iPSC_WT_AH017_rep3_R2.fastq.gz
ChIP_H3K4me1_Ery_iPSC_WT_SBAD02_rep1_R1.fastq.gz
ChIP_H3K4me1_Ery_iPSC_WT_SBAD02_rep1_R2.fastq.gz
ChIP_H3K4me1_Ery_iPSC_WT_SBAD02_rep2_R1.fastq.gz
ChIP_H3K4me1_Ery_iPSC_WT_SBAD02_rep2_R2.fastq.gz
ChIP_H3K4me1_Ery_iPSC_WT_SBAD02_rep3_R1.fastq.gz
ChIP_H3K4me1_Ery_iPSC_WT_SBAD02_rep3_R2.fastq.gz
ChIP_H3K4me3_Ery_iPSC_C-SNV_LA01_rep1_R1.fastq.gz
ChIP_H3K4me3_Ery_iPSC_C-SNV_LA01_rep1_R2.fastq.gz
ChIP_H3K4me3_Ery_iPSC_C-SNV_LA06_rep1_R1.fastq.gz
ChIP_H3K4me3_Ery_iPSC_C-SNV_LA06_rep1_R2.fastq.gz
ChIP_H3K4me3_Ery_iPSC_C-SNV_LA13_rep1_R1.fastq.gz
ChIP_H3K4me3_Ery_iPSC_C-SNV_LA13_rep1_R2.fastq.gz
ChIP_H3K4me3_Ery_iPSC_INV_Clone1_rep1_R1.fastq.gz
ChIP_H3K4me3_Ery_iPSC_INV_Clone1_rep1_R2.fastq.gz
ChIP_H3K4me3_Ery_iPSC_INV_Clone2_rep1_R1.fastq.gz
ChIP_H3K4me3_Ery_iPSC_INV_Clone2_rep1_R2.fastq.gz
ChIP_H3K4me3_Ery_iPSC_Vas_Clone1_rep1_R1.fastq.gz
ChIP_H3K4me3_Ery_iPSC_Vas_Clone1_rep1_R2.fastq.gz
ChIP_H3K4me3_Ery_iPSC_Vas_Clone2_rep1_R1.fastq.gz
ChIP_H3K4me3_Ery_iPSC_Vas_Clone2_rep1_R2.fastq.gz
ChIP_H3K4me3_Ery_iPSC_Vas_Clone3_rep1_R1.fastq.gz
ChIP_H3K4me3_Ery_iPSC_Vas_Clone3_rep1_R2.fastq.gz
ChIP_H3K4me3_Ery_iPSC_Vs_Clone1_rep1_R1.fastq.gz
ChIP_H3K4me3_Ery_iPSC_Vs_Clone1_rep1_R2.fastq.gz
ChIP_H3K4me3_Ery_iPSC_Vs_Clone2_rep1_R1.fastq.gz
ChIP_H3K4me3_Ery_iPSC_Vs_Clone2_rep1_R2.fastq.gz
ChIP_H3K4me3_Ery_iPSC_Vs_Clone3_rep1_R1.fastq.gz
ChIP_H3K4me3_Ery_iPSC_Vs_Clone3_rep1_R2.fastq.gz
ChIP_H3K4me3_Ery_iPSC_WT_AH017_rep1_R1.fastq.gz
ChIP_H3K4me3_Ery_iPSC_WT_AH017_rep1_R2.fastq.gz
ChIP_H3K4me3_Ery_iPSC_WT_AH017_rep2_R1.fastq.gz
ChIP_H3K4me3_Ery_iPSC_WT_AH017_rep2_R2.fastq.gz
ChIP_H3K4me3_Ery_iPSC_WT_AH017_rep3_R1.fastq.gz
ChIP_H3K4me3_Ery_iPSC_WT_AH017_rep3_R2.fastq.gz
ChIP_H3K4me3_Ery_iPSC_WT_SBAD02_rep1_R1.fastq.gz
ChIP_H3K4me3_Ery_iPSC_WT_SBAD02_rep1_R2.fastq.gz
ChIP_H3K4me3_Ery_iPSC_WT_SBAD02_rep2_R1.fastq.gz
ChIP_H3K4me3_Ery_iPSC_WT_SBAD02_rep2_R2.fastq.gz
ChIP_H3K4me3_Ery_iPSC_WT_SBAD02_rep3_R1.fastq.gz
ChIP_H3K4me3_Ery_iPSC_WT_SBAD02_rep3_R2.fastq.gz
ChIP_RNAPII_Ery_iPSC_C-SNV_LA01_rep1_R1.fastq.gz
ChIP_RNAPII_Ery_iPSC_C-SNV_LA01_rep1_R2.fastq.gz
ChIP_RNAPII_Ery_iPSC_C-SNV_LA06_rep1_R1.fastq.gz
ChIP_RNAPII_Ery_iPSC_C-SNV_LA06_rep1_R2.fastq.gz
ChIP_RNAPII_Ery_iPSC_C-SNV_LA13_rep1_R1.fastq.gz
ChIP_RNAPII_Ery_iPSC_C-SNV_LA13_rep1_R2.fastq.gz
ChIP_RNAPII_Ery_iPSC_INV_Clone1_rep1_R1.fastq.gz
ChIP_RNAPII_Ery_iPSC_INV_Clone1_rep1_R2.fastq.gz
ChIP_RNAPII_Ery_iPSC_INV_Clone2_rep1_R1.fastq.gz
ChIP_RNAPII_Ery_iPSC_INV_Clone2_rep1_R2.fastq.gz
ChIP_RNAPII_Ery_iPSC_Vs_Clone1_rep1_R1.fastq.gz
ChIP_RNAPII_Ery_iPSC_Vs_Clone1_rep1_R2.fastq.gz
ChIP_RNAPII_Ery_iPSC_Vs_Clone2_rep1_R1.fastq.gz
ChIP_RNAPII_Ery_iPSC_Vs_Clone2_rep1_R2.fastq.gz
ChIP_RNAPII_Ery_iPSC_Vs_Clone3_rep1_R1.fastq.gz
ChIP_RNAPII_Ery_iPSC_Vs_Clone3_rep1_R2.fastq.gz
ChIP_RNAPII_Ery_iPSC_WT_AH017_rep1_R1.fastq.gz
ChIP_RNAPII_Ery_iPSC_WT_AH017_rep1_R2.fastq.gz
ChIP_RNAPII_Ery_iPSC_WT_AH017_rep2_R1.fastq.gz
ChIP_RNAPII_Ery_iPSC_WT_AH017_rep2_R2.fastq.gz

ChIP_RNAPII_Ery_iPSC_WT_AH017_rep3_R1.fastq.gz
ChIP_RNAPII_Ery_iPSC_WT_AH017_rep3_R2.fastq.gz
ChIP_RNAPII_Ery_iPSC_WT_SBAD02_rep1_R1.fastq.gz
ChIP_RNAPII_Ery_iPSC_WT_SBAD02_rep1_R2.fastq.gz
ChIP_RNAPII_Ery_iPSC_WT_SBAD02_rep2_R1.fastq.gz
ChIP_RNAPII_Ery_iPSC_WT_SBAD02_rep2_R2.fastq.gz
ChIP_RNAPII_Ery_iPSC_WT_SBAD02_rep3_R1.fastq.gz
ChIP_RNAPII_Ery_iPSC_WT_SBAD02_rep3_R2.fastq.gz

Genome browser session
(e.g. UCSC)

none

Methodology

Replicates

To characterise the epigenetic landscape and identify chromatin modifications, ChIP-seq was carried out at day 21 of hiPSC erythroid differentiation. ChIP was performed for enhancer (H3K4me1), promoter (H3K4me3) and active transcription (H3K27ac) marks, as well as for the boundary protein CTCF and RNA polymerase II (RNAPII). All ChIPs were performed on the same material simultaneously and a 5% input was stored prior to immunoprecipitation as a control. Lines: LA01, LA06, LA13 (homozygous C-SNV allele); Vas_Clone1, Vas_Clone2, Vas_Clone3 (promoter insertion in anti-sense orientation, parental line SB-AD2-01); Vs_Clone1, Vs_Clone2, Vs_Clone3 (promoter insertion in sense orientation, parental line SB-AD2-01); INV_Clone1, INV_Clone2, (C-SNV promoter inversion, parental line LA01); AH017-13 (abbr. AH017) wild type line; SB-AD2-01 (abbr. SBAD02) wild type line; Further information is available on the GEO page associated with this data set.

Samples:

ChIP_CTCF_Ery_iPSC_C-SNV_LA01_rep1
ChIP_CTCF_Ery_iPSC_C-SNV_LA06_rep1
ChIP_CTCF_Ery_iPSC_C-SNV_LA13_rep1
ChIP_CTCF_Ery_iPSC_WT_AH017_rep1
ChIP_CTCF_Ery_iPSC_WT_AH017_rep2
ChIP_CTCF_Ery_iPSC_WT_AH017_rep3
ChIP_H3K27ac_Ery_iPSC_C-SNV_LA01_rep1
ChIP_H3K27ac_Ery_iPSC_C-SNV_LA06_rep1
ChIP_H3K27ac_Ery_iPSC_C-SNV_LA13_rep1
ChIP_H3K27ac_Ery_iPSC_Vas_Clone1_rep1
ChIP_H3K27ac_Ery_iPSC_Vas_Clone2_rep1
ChIP_H3K27ac_Ery_iPSC_Vas_Clone3_rep1
ChIP_H3K27ac_Ery_iPSC_Vs_Clone1_rep1
ChIP_H3K27ac_Ery_iPSC_Vs_Clone2_rep1
ChIP_H3K27ac_Ery_iPSC_Vs_Clone3_rep1
ChIP_H3K27ac_Ery_iPSC_WT_AH017_rep1
ChIP_H3K27ac_Ery_iPSC_WT_AH017_rep2
ChIP_H3K27ac_Ery_iPSC_WT_AH017_rep3
ChIP_H3K27ac_Ery_iPSC_WT_SBAD02_rep1
ChIP_H3K27ac_Ery_iPSC_WT_SBAD02_rep2
ChIP_H3K27ac_Ery_iPSC_WT_SBAD02_rep3
ChIP_H3K4me1_Ery_iPSC_C-SNV_LA01_rep1
ChIP_H3K4me1_Ery_iPSC_C-SNV_LA06_rep1
ChIP_H3K4me1_Ery_iPSC_C-SNV_LA13_rep1
ChIP_H3K4me1_Ery_iPSC_Vas_Clone1_rep1
ChIP_H3K4me1_Ery_iPSC_Vas_Clone2_rep1
ChIP_H3K4me1_Ery_iPSC_Vas_Clone3_rep1
ChIP_H3K4me1_Ery_iPSC_Vs_Clone1_rep1
ChIP_H3K4me1_Ery_iPSC_Vs_Clone2_rep1
ChIP_H3K4me1_Ery_iPSC_Vs_Clone3_rep1
ChIP_H3K4me1_Ery_iPSC_WT_AH017_rep1
ChIP_H3K4me1_Ery_iPSC_WT_AH017_rep2
ChIP_H3K4me1_Ery_iPSC_WT_AH017_rep3
ChIP_H3K4me1_Ery_iPSC_WT_SBAD02_rep1
ChIP_H3K4me1_Ery_iPSC_WT_SBAD02_rep2
ChIP_H3K4me1_Ery_iPSC_WT_SBAD02_rep3
ChIP_H3K4me3_Ery_iPSC_C-SNV_LA01_rep1
ChIP_H3K4me3_Ery_iPSC_C-SNV_LA06_rep1
ChIP_H3K4me3_Ery_iPSC_C-SNV_LA13_rep1
ChIP_H3K4me3_Ery_iPSC_INV_Clone1_rep1
ChIP_H3K4me3_Ery_iPSC_INV_Clone2_rep1
ChIP_H3K4me3_Ery_iPSC_Vas_Clone1_rep1
ChIP_H3K4me3_Ery_iPSC_Vas_Clone2_rep1
ChIP_H3K4me3_Ery_iPSC_Vas_Clone3_rep1

	ChIP_H3K4me3_Ery_iPSC_Vs_Clone1_rep1<br>ChIP_H3K4me3_Ery_iPSC_Vs_Clone2_rep1<br>ChIP_H3K4me3_Ery_iPSC_Vs_Clone3_rep1<br>ChIP_H3K4me3_Ery_iPSC_WT_AH017_rep1<br>ChIP_H3K4me3_Ery_iPSC_WT_AH017_rep2<br>ChIP_H3K4me3_Ery_iPSC_WT_AH017_rep3<br>ChIP_H3K4me3_Ery_iPSC_WT_SBAD02_rep1<br>ChIP_H3K4me3_Ery_iPSC_WT_SBAD02_rep2<br>ChIP_H3K4me3_Ery_iPSC_WT_SBAD02_rep3<br>ChIP_RNAPII_Ery_iPSC_C-SNV_LA01_rep1<br>ChIP_RNAPII_Ery_iPSC_C-SNV_LA06_rep1<br>ChIP_RNAPII_Ery_iPSC_C-SNV_LA13_rep1<br>ChIP_RNAPII_Ery_iPSC_INV_Clone1_rep1<br>ChIP_RNAPII_Ery_iPSC_INV_Clone2_rep1<br>ChIP_RNAPII_Ery_iPSC_Vs_Clone1_rep1<br>ChIP_RNAPII_Ery_iPSC_Vs_Clone2_rep1<br>ChIP_RNAPII_Ery_iPSC_Vs_Clone3_rep1<br>ChIP_RNAPII_Ery_iPSC_WT_AH017_rep1<br>ChIP_RNAPII_Ery_iPSC_WT_AH017_rep2<br>ChIP_RNAPII_Ery_iPSC_WT_AH017_rep3<br>ChIP_RNAPII_Ery_iPSC_WT_SBAD02_rep1<br>ChIP_RNAPII_Ery_iPSC_WT_SBAD02_rep2<br>ChIP_RNAPII_Ery_iPSC_WT_SBAD02_rep3
Sequencing depth	All replicates have > 15M reads. Paired-end sequencing 40bp reads.
Antibodies	RNA Polymerase II N-20 antibody SC-899 (Santa Cruz Biotechnology) 6µg per 1ml of chromatin from 1x10^7 cells;<br>Monoclonal anti-acetyl-Histone H3 (Lys27) antibody 17-683 (Merck Millipore) 2µg per 1ml of chromatin from 1x10^7 cells;<br>Polyclonal anti-trimethyl-Histone H3 (Lys4) antibody 07-473 (Merck Millipore) 1µg per 1ml of chromatin from 1x10^7 cells;<br>Polyclonal antimonomethyl-Histone H3 (Lys4) antibody ab195391 (Abcam) 4µg per 1ml of chromatin from 1x10^7 cells;<br>Polyclonal anti-GATA1 antibody ab11852 (Abcam) 8µg per 1ml of chromatin from 1x10^7 cells;<br>Polyclonal anti-CTCF antibody 07-729 (Merck Millipore) 10µl serum per 1ml of chromatin from 1x10^7 cells<br>Polyclonal anti-KLF1 antibody was kindly provided by the Perkins Laboratory, Translational Research Institute, Brisbane, Australia
Peak calling parameters	none
Data quality	ChIP enrichment was verified by qPCR prior to sequencing. Quality of sequencing was verified by FastQC.
Software	Software packages that were used for the analysis include FASTQC 0.11.9, Bowtie 2.3.2, Samtools 0.1.19, Bedtools 2.25.0, Deeptools 2.2.2. For ChIP-seq, data analysis was performed using an in-house pipeline: https://github.com/Hughes-Genome-Group/NGseqBasic/releases.

Flow Cytometry

Plots

Confirm that:

- [x] The axis labels state the marker and fluorochrome used (e.g. CD4-FITC).
- [x] The axis scales are clearly visible. Include numbers along axes only for bottom left plot of group (a 'group' is an analysis of identical markers).
- [x] All plots are contour plots with outliers or pseudocolor plots.
- [x] A numerical value for number of cells or percentage (with statistics) is provided.

Methodology

Sample preparation	Flow cytometry analysis was performed on 2x10^5 cells resuspended in 200µl of 2% bovine serum albumin (SIGMA) in phosphate buffered saline and labelled for 20 minutes at 4oC with 1:200 dilution of fluorescein isothiocyanate (FITC) conjugated anti-CD71 (BD Biosciences; 555536) and 1:200 dilution of phycoerythrin (PE) conjugated anti-CD235a (BD Biosciences; 340947). After staining cells were harvested and resuspended in PBS containing 0.02% Hoechst 33258 pentahydrate nucleic acid stain (Invitrogen).
Instrument	Attune NxT Flow Cytometer
Software	FlowJo 10.6.2
Cell population abundance	All cultures were >90% CD71+ CD235a+ double positive (with >94% CD235a+) erythroid cells.

Gating strategy

Side Scatter Area vs Forward Scatter Area to select cells > >> Forward Scatter Hight vs Forward Scatter Area > >> FL 6 (Fixable Violet) Area vs Forward Scatter Area to select viable cells > > > FL 2 Area: CD235a-PE vs FL 1 Area: CD71-FITC

☒ Tick this box to confirm that a figure exemplifying the gating strategy is provided in the Supplementary Information.
